# Polymer Length Governs DNA Adsorption Dynamics on Mineral Surfaces

**DOI:** 10.1021/acs.est.5c08180

**Published:** 2025-09-18

**Authors:** Veer Vikram Singh, Naresh Kumar, Richard L. Kimber, Ákos Weiser, Ron Pinhasi, Stephan M. Kraemer

**Affiliations:** † Department of Environmental Geosciences, Centre for Microbiology and Environmental Systems Science, 27258 University of Vienna, Josef-Holaubek-Platz 2, 1090 Vienna, Austria; ‡ Doctoral School in Microbiology and Environmental Science, University of Vienna, Josef-Holaubek-Platz 2, 1090 Vienna, Austria; § Soil Chemistry, 4508 Wageningen University and Research, Droevendaalsesteeg 3, 6708 PB Wageningen, The Netherlands; ∥ Department of Earth and Environmental Sciences, 5292 The University of Manchester, M13 9PL Manchester, U.K.; ⊥ Department of Evolutionary Anthropology, University of Vienna, Djerassiplatz 1, 1030 Vienna, Austria

**Keywords:** eDNA, competitive adsorption, DNA persistence, DNA biogeochemical cycling, aDNA

## Abstract

DNA adsorption onto mineral surfaces plays a crucial role in controlling its biogeochemical cycling, environmental stability, and accessibility for diverse environmental DNA (eDNA) applications. While eDNA exists in a wide range of polymer lengths, a limited understanding of how DNA polymer length influences adsorption and competition on mineral surfaces hinders accurate interpretations of its mobility and persistence in natural systems. Here, we address this knowledge gap by investigating the role of DNA polymer length (99 bp to ∼20,000 bp) on interactions with selected environmentally relevant minerals, including Fe­(III)-(oxyhydr)­oxides (goethite, 2-line ferrihydrite), clays (kaolinite, montmorillonite) and hydroxyapatite. Controlled batch experiments at neutral pH show that adsorption from model uniform DNA solutions increases with increasing polymer length for Fe­(III)-(oxyhydr)­oxides and clays, with the reverse trend observed for hydroxyapatite. During competitive adsorption experiments (using 99 and 2000 bp DNA), the order of addition influenced the extent of adsorption. However, under simultaneous addition—closely reflecting natural environmental conditions, where both polymers compete for binding sites—shorter DNA polymers exhibited preferential adsorption across all minerals. We hypothesize that this preferential adsorption may contribute toward the enhanced environmental persistence of shorter DNA polymers, including the exclusive preservation of small DNA polymers (<100 bp) over long time scales. These findings underscore the critical role of polymer length in DNA adsorption and provide a basis for mechanistic insights into the factors influencing its preservation and fate in natural environments with implications for a range of DNA-based technologies.

## Graphical abstract

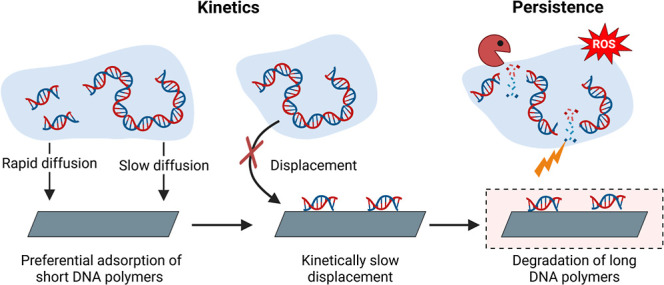

## 1 Introduction

The adsorption, retention and preservation of DNA on mineral surfaces have widespread implications for a diverse array of nucleic acid-based applications, including biodiversity monitoring,^1,2^ paleogenetics,^3–5^ therapeutic gene delivery,^6,7^ and forensics,^8^ as well as for environmental processes such as phosphorus cycling,^9^ and horizontal gene transfer.^10,11^ Environmental minerals offer high adsorption potential owing to their abundance, high specific surface areas, chemical reactivity and structural diversity.^12^ Adsorption of environmental DNA (eDNA) onto mineral surfaces can protect DNA from a range of environmental degradation mechanisms, including enzymatic hydrolysis^13–16^ and UV radiation,^17^ enhancing its persistence and functional availability. Even under extremely high nuclease activity, mineral-bound DNA has been shown to retain biological functionality and facilitate natural transformation,^18^ potentially influencing horizontal gene transfer and the propagation of antibiotic-resistant genes in the environment.^11,19^ The recent recovery of two-million-year-old ancient DNA (aDNA) from sediments illustrates the possible role of mineral surfaces in preserving genetic material over geological time scales, highlighting their potential as a huge and largely untapped genetic repository.^20^ Given the critical role of adsorption in determining DNA fate, it is essential to understand factors influencing its interaction with mineral surfaces. While numerous studies have investigated the influence of solution physicochemical parameters, including pH, ionic strength, and ion types, on the rate, extent and mechanisms of DNA adsorption,^21–25^ the role of molecular characteristics of DNA itself, particularly polymer length, in governing adsorption onto mineral surfaces remains poorly understood.

In living organisms, DNA polymer length ranges from a few thousand base pairs (bp) in the mitochondrial genome to a few hundred million bp in the nuclear genome.^26^ Once released into the environment, swift decay by various physicochemical pathways yields a heterogeneous distribution of polymer lengths coexisting in environmental systems.^27,28^ With prolonged exposure to degradation, highly fragmented short DNA polymers dominate in the environment,^29–31^ eventually resulting in exclusive preservation of small sequences (typically <100 bp) over extended time scales as commonly reported in aDNA studies.^20,32–34^ Similar to other polyelectrolytes on charged surfaces, DNA polymers of varying lengths are likely to exhibit distinct adsorption affinities and spatial organizations on mineral surfaces, potentially competing for adsorption sites.^35^ Such competition may drive preferential adsorption of specific size fractions or displacement of previously adsorbed DNA, significantly impacting the fate of DNA in the environment. The potential of eDNA in ecology and conservation applications, as well as of aDNA in anthropology, primarily relies on the efficiency of genetic information retrieval from these highly fragmented DNA polymers.^32,34,36^ Therefore, understanding how polymer length influences DNA adsorption is critical for improving the acquisition and interpretation of reliable genetic data from environmental samples. Additionally, a mechanistic understanding of adsorption dynamics across different minerals can help design targeted sampling approaches to retrieve DNA from mineral surfaces that have high preservation potentials and significantly improve the efficiency of these applications. Despite its significance, the influence of DNA polymer length on adsorption behavior—particularly for environmentally relevant short fragments (<100 bp)—remains poorly understood.

Most existing research utilizes relatively long DNA polymers (often thousands to tens of thousands bp) and reports varying trends in adsorption (typically expressed as the DNA mass adsorbed per unit mineral mass or surface area) with polymer length.^37–41^ These studies often use nonuniform DNA (with a broad polymer length distribution) instead of uniform DNA (polymers with identical length), making it challenging to evaluate size-dependent effects.^13,22,42^ Additionally, often these studies use polymers with varying conformations (*e.g.*, supercoiled plasmid *vs* linear chromosomal), which may add complexity to the interpretation of the effect of polymer length on DNA adsorption.^38,39,41^ Finally, most DNA adsorption studies focus on single mineral groups, limiting cross-mineral comparisons and understanding of rates, extents and mechanisms under identical physicochemical conditions.

The present study provides a systematic understanding of the polymer length (expressed hereafter in bp) influences adsorption to various minerals under different geochemical conditions. Using PCR-synthesized uniform DNA (99, 400, 1000, 2000, and 4000 bp) and nonuniform genomic DNA (median length ∼20,000 bp), we investigated adsorption onto a diverse set of environmentally and biologically relevant minerals: goethite and ferrihydrite (common Fe­(III)-(oxyhydr)­oxides in soil and sediments), kaolinite, montmorillonite (common nonswelling and swelling clays, respectively) and hydroxyapatite (bone mineral). The DNA polymers used in this study were linear, as PCR amplification yields linear fragments, and the genomic DNA extracted from salmon sperm cells primarily consists of chromosomal DNA, which is inherently linear.^38,39^ We conducted a series of controlled batch experiments to study kinetics, isotherms, and the effects of pH and polymer length of DNA on adsorption to these diverse minerals under identical and comparable experimental parameters. We also explored the relative and competitive surface affinity of DNA of varying polymer lengths using 99 and 2000 bp uniform DNA over time. Our results highlight the importance of DNA polymer length in exerting control over persistence and competitive mobilization in the environment, with potentially significant implications for DNA biogeochemical cycling and DNA-based technologies such as biodiversity monitoring.

## 2 Materials and Methods

### 2.1 Chemicals and Supplies

All chemicals and supplies used were of analytical grade (Section S1). We used nuclease-free disposables (tubes, pipet tips) and autoclaved glassware (121 °C, 60 min). pH buffers (acetate for pH 5, 2-(*N*-morpholino)­ethanesulfonic acid (MES) for pH 6, 4-(2-hydroxyethyl)-1-piperazineethanesulfonic acid (HEPES) for pH 7–8, and sodium tetraborate for pH 9) were prepared using ultrapure water (resistivity ≥18.2 MΩ·cm), filtered using 0.1 μm low protein binding sterile filters (Merck Millipore Ltd.), autoclaved, and aliquoted before storage at 4 °C (for <1 week) or at −20 °C (if stored for >1 week). dsDNA polymers of length 99, 400, 1000, 2000, and 4000 bp were synthesized by polymerase chain reaction (PCR) using plasmid pBR322 (Thermo Fisher Scientific, USA) as a template, purified using MinElute PCR Purification Kit (Qiagen, USA), eluted into desired pH buffers, and stored at −20 °C until use. PCR parameters, sequences and physicochemical properties of DNA polymers are provided in Section S2. DNA sodium salt from salmon testes, referred to as genomic DNA (gDNA) hereafter, with a median polymer length of ∼20,000 bp (Sigma-Aldrich, USA) was used to prepare stock solutions by dissolving the gDNA sodium salt in desired pH buffers, which were used within 24 h. DNA polymer lengths were analyzed on Agilent TapeStation 4150 using various ScreenTapes as per the manufacturer’s instructions (Figure S1).

### 2.2 Mineral Synthesis and Characterization

Detailed procedures for mineral synthesis, purification, and characterization are provided in Section S3. Briefly, goethite and 2-line ferrihydrite were synthesized using the chemical precipitation method of Schwertmann and Cornell^43^ as previously described in Kumar et al. 2018.^44^ Biomimetic hydroxyapatite was synthesized by an alkaline precipitation method of Li et al. 2020.^45^ The precipitated Fe­(III)-(oxyhydr)­oxides and hydroxyapatite were thoroughly washed with ultrapure water (5–7 times) to remove any undesired ions. Kaolinite (KGa-1b) and montmorillonite (SWy-3) were purchased from the source clay repositories of the Clay Mineral Society, USA. The clay fraction with <2 μm hydrodynamic diameter was separated by gravitative settling, Na^+^ saturated and dialyzed until the conductivity of the solution reached <1 μS/cm to remove excess ions (further details in Supporting Information). All minerals were freeze-dried, ground, and stored in amber glass tubes either at 4 °C (for goethite, kaolinite, and montmorillonite) or at −80 °C (ferrihydrite and hydroxyapatite) to minimize mineral transformation. Purity and specific surface area (SSA) of each mineral were determined using X-ray diffraction analysis and N_2_-BET analysis, respectively.

Mineral stocks for experiments were freshly prepared by suspending in ultrapure water with continuous stirring (300 rpm, 24 h) for mineral-surface hydration.^24^ To achieve better particle dispersion, mineral stocks were ultrasonicated for 3 min at 35 kHz (Bandelin Sonorex RK 100, Bandelin Electronics, Germany) immediately before experiments. All experiments were performed in triplicates run in parallel in the dark at 20 °C in 2 mL protein LoBind tubes, with samples agitated at 30 rpm on an overhead shaker. Samples were centrifuged at 20,000*g* for 20 min (40 min for montmorillonite), followed by supernatant collection and immediate DNA analyses.

### 2.3 Experimental Setup

#### 2.3.1 Adsorption Kinetics, Isotherms and Effect of pH on Adsorption

(i) Adsorption kinetics were studied by mixing gDNA (50 ng/μL) with mineral suspensions (up to 1000 μL) at pH 7, followed by regular sampling of 20 μL aliquots from 30 s up to 24 h. Based on results from adsorption kinetics, a 6 h time frame was chosen for adsorption isotherms and pH-dependent adsorption experiments. (ii) To evaluate the effect of pH on DNA adsorption capacity, gDNA (80 ng/μL) was incubated with minerals (up to 100 μL) at pH 5, 6, 7, 8, and 9. (iii) Adsorption isotherms were obtained by incubating gDNA and 99 bp DNA with minerals at DNA concentrations ranging from 5–150 ng/μL (up to 100 μL) at pH 7. The mineral loadings (in mg/mL) for these experiments were chosen at 2 for goethite and ferrihydrite, 4 for kaolinite, 1 for montmorillonite, and 1.5 for hydroxyapatite.

#### 2.3.2 Effect of DNA Polymer Length on Adsorption and Competitive Adsorption

To evaluate the effect of DNA polymer length on adsorption, 20 ng/μL DNA polymers (99, 400, 1000, 4000 and ∼20,000 bp (gDNA)) were incubated with minerals (up to 100 μL) for 6 h at pH 7. An equal mass concentration of DNA polymers was applied for these experiments, ensuring an equal number of overall phosphate groups for each DNA fragment length. Competitive adsorption was studied through three sets of experiments: (i) 99 and 2000 bp DNA were added simultaneously to the mineral suspension, the influence of the order of addition was studied by (ii) first adding 99 bp DNA followed by 2000 bp DNA 30 min later, or (iii) by first adding 2000 bp DNA and then 99 bp DNA. Additionally, two control adsorption experiments using only 99 bp or 2000 bp under identical conditions were run in parallel. Each DNA polymer was added to a final 15 ng/μL concentration. After mixing both DNA polymers with the minerals (up to 400 μL), 20 μL aliquots were collected for analysis at 30 min, 6 h, 1 day, 7 days and 14 days. Lower mineral loadings (mg/mL) of 0.3 for goethite, 1 for ferrihydrite, 1.2 for kaolinite, 0.5 for montmorillonite, and 0.15 for hydroxyapatite were used to evaluate the effect of DNA polymer length on adsorption and competitive adsorption.

The zeta potential of minerals with or without adsorbed DNA was measured on Anton Paar Litesizer 500. For each measurement, 2.5 ng/μL of either 99 bp DNA or gDNA was incubated with the minerals in 500 μL solution volumes, with mineral loadings identical to those used in the competitive adsorption experiments.

### 2.4 DNA Analyses

The stock concentrations of DNA polymers were determined by measuring UV absorbance at 260 nm on a Tecan Infinite Pro 200 spectrophotometer (Tecan) with NanoQuant plate using an extinction coefficient of 0.020 (ng/μL)^−1^ cm^–1^. Due to higher sensitivity, fluorometric assays (Qubit 1× dsDNA BR, Thermo Fisher) were used to quantify DNA in experimental supernatants using a polymer-specific calibration curve prepared by serial dilution of stock DNA (100 ng/μL). The assays were performed by mixing 5 μL of sample with 195 μL of 1× BR buffer in Qubit assay tubes and analyzed on a Qubit 4 fluorometer. The Limit of quantification (LOQ) was calculated as described by Armbruster and Pry^46^ using eq 1, where μ_blank_ represents the mean of the blank measurements, while σ_blank_ and σ_standard_ represent the standard deviations of the blank and a low-concentration sample, respectively. The LOQ ranged between 0.11–0.15 ng/μL for all DNA polymers.

1 $$LOQ=\left(\left(\mu_{blank}+{1.645\times \sigma }_{blank}\right)+{1.645\times \sigma }_{standard}\right)$$

The amount of DNA adsorbed to the mineral surfaces was calculated by depletion from the solution and normalized to the surface area of minerals using eq 2. Here, DNA_i_ and DNA_f_ are the initial and final DNA concentration (ng/μL), *C*_mineral_ is the mineral loading (mg/mL), and SSA (m^2^/g) is the specific surface area of the respective mineral.

2 $$\Gamma_{DNA}\;\left(ng/{cm}^{2}\right)=\frac{\left({DNA}_{i}-{DNA}_{f}\right)\times 100}{SSA\times C_{mineral}}$$

We found that the mass fraction of 99 and 2000 bp DNA from mixed solutions correlated linearly with their relative peak intensities on TapeStation (D5000 screen tape). A calibration curve was created by preparing solutions of varying mass fractions of 99 and 2000 bp DNA and measuring their corresponding relative peak intensities. The absolute concentrations of each DNA polymer during competitive adsorption experiments were determined using linear regression to calibration curves (Figure S4). Although the free and surface-bound eDNA concentrations in natural environments are typically below the experimental range used,^29^ the choice of higher concentrations aligns with previous studies and was necessary to achieve plateau adsorption, allowing comparison of maximum adsorption capacities across minerals.^37–41^ All statistical analyses were performed using GraphPad Prism 10.4.1.

## 3 Results and Discussion

### 3.1 Adsorption Kinetics

We first investigated the kinetics of DNA adsorption onto various mineral surfaces using gDNA at an initial concentration of 50 ng/μL at pH 7 (Figure 1a). Adsorption of gDNA was rapid initially, with more than 90% gDNA adsorbed within 1 h of addition for all minerals, followed by a slower increase in total adsorption up to 24 h for goethite, kaolinite and hydroxyapatite. A similar two-step adsorption has been previously reported for DNA on montmorillonite,^22,47^ goethite,^24,48^ hydroxyapatite,^49^ and for other polyelectrolytes on charged surfaces.^50–53^ Interestingly, DNA adsorption to ferrihydrite and montmorillonite did not change significantly from 1 to 24 h (paired *t*-test, *p* > 0.05) while adsorption to hydroxyapatite increased very little.

**Figure 1:**
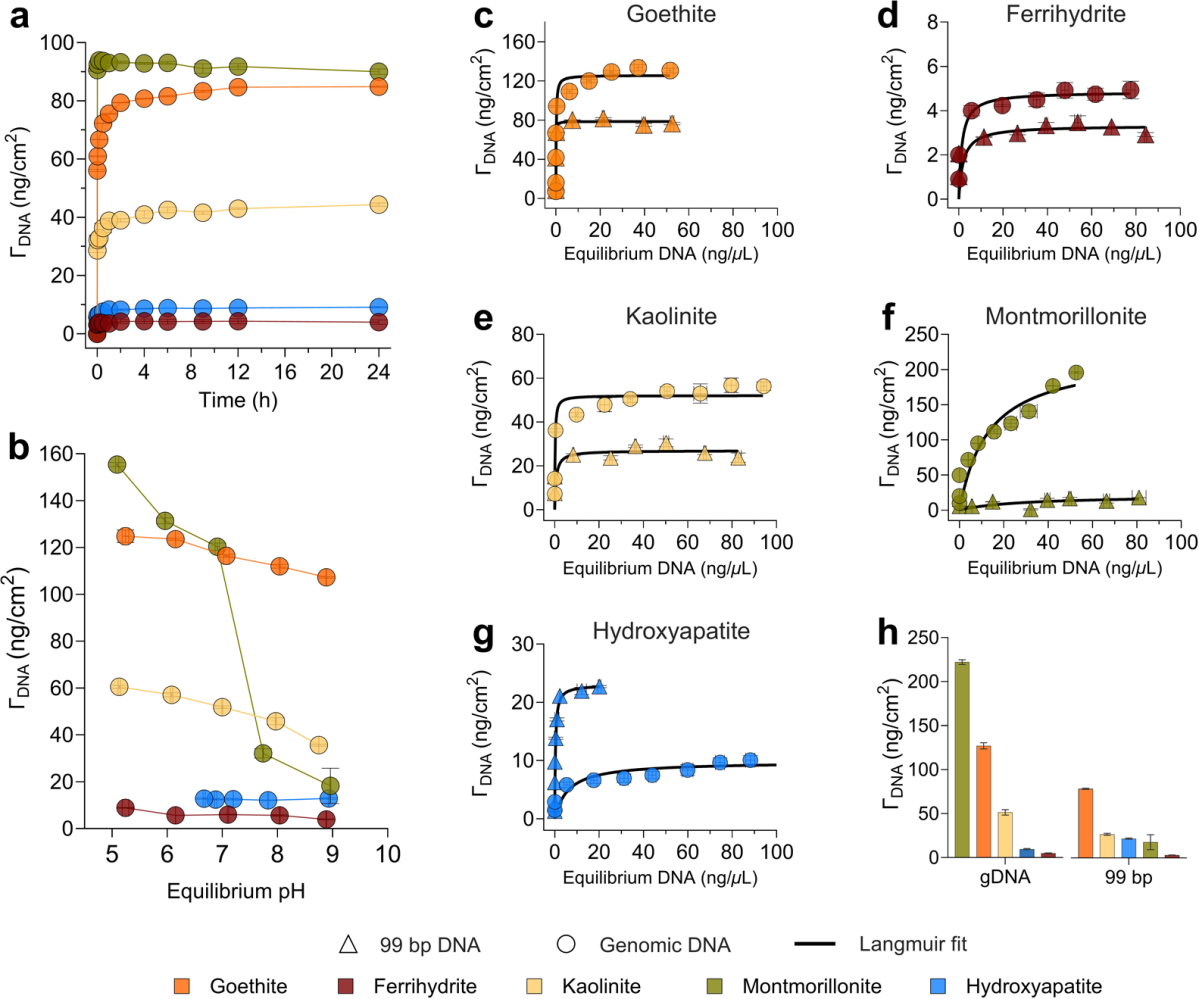
(a) Adsorption kinetics of gDNA with an initial DNA concentration of 50 ng/μL at pH 7. (b) pH-dependent adsorption of gDNA with an initial DNA concentration of 80 ng/μL between pH 5 and 9 for 6 h of incubation. (c–g) Adsorption isotherms of 99 bp and gDNA at initial concentrations ranging between 5 and 150 ng/μL (depending on mineral) at pH 7 for 6 h incubation. (h) Comparison of maximum adsorption capacities of 99 bp and gDNA to minerals derived from Langmuir fit to the adsorption isotherms. All experiments were conducted in a background solution of 30 mM NaCl with 3 mM buffer species (see Section 2.1). During kinetics and isotherms, pH remained within ±0.1 units of the initial pH for all minerals except hydroxyapatite (final pH 7.2). Final pH values are shown in (b). Data points and error bars represent the mean and standard deviation of triplicates run in parallel.

It has been demonstrated for polyelectrolytes^51,52^ and also suggested for DNA^54^ that the initial rapid phase of adsorption is governed by the diffusion from the bulk solution to the mineral surface, followed by surface binding. DNA binds to the mineral surfaces through various mechanisms depending on physicochemical factors, including mineral type, solution chemistry (ionic strength, ion type, *etc.*), pH, and DNA structure (single *vs* double-stranded). dsDNA polymers, as used in this study, primarily bind through phosphate groups exposed on their backbone.^24^ Binding mechanisms include ligand exchange, where the DNA phosphate group displaces the hydroxyl group on mineral surfaces, forming a monodentate inner-sphere complex. This includes Fe–O–P bond formation on Fe­(III)-(oxyhydr)­oxide surfaces,^55–58^ Si–O–P bonds at edges, and Al–O–P bonds on the octahedral surfaces and clay edges.^41,54,59^ Electrostatic interactions may also play a significant role, considering the permanent and/or variable charge of the mineral surfaces and the charge of the surface complexes, including ternary surface complexes with bridging cations such as calcium or the charge of nonbonding phosphate groups of adsorbed polymers.^21,49,60^ Na^+^ ions may screen the negative charge on mineral surfaces and the DNA backbone, facilitating their mutual approach and binding.^60,61^ However, in natural environments, multivalent cations (*e.g.*, Ca^2+^, Mg^2+^) can facilitate cation bridging and induce DNA compaction, in addition to charge screening, leading to a greater extent of DNA adsorption compared to the monovalent cation (Na^+^).^22,62^ Additional interactions, such as hydrogen bonding between oxygen atoms of DNA phosphates to surface hydroxyl groups and van der Waals interactions, may further contribute to DNA–mineral binding, although these contributions are expected to be smaller compared to electrostatic interactions and inner-sphere complexation.^24^

As the surface coverage increases, transport-controlled kinetics becomes less influential, and further adsorption becomes increasingly constrained by the availability of free adsorption sites and/or steric hindrance from previously adsorbed polymers.^51,63,64^ Adsorbed polymers may also undergo conformational changes upon adsorption (*e.g.*, change from B to Z form),^13^ or rearrangements, such as relaxation on the charged surfaces.^48,65,66^ However, these processes typically occur much slower than the initial transport-controlled adsorption kinetics, resulting in the slow adsorption phase observed after the initial fast adsorption stage.^50–52^

### 3.2 Effect of Solution pH on DNA Adsorption

To evaluate the effect of pH on DNA adsorption, 80 ng/μL of gDNA was incubated with minerals at pH 5, 6, 7, 8, and 9 for 6 h. This reaction time ensured >94% gDNA adsorption relative to the adsorption observed at 24 h for all minerals (Figure 1a). Adsorption (ng/cm^2^) systematically decreased with increasing pH from 5 to 9 for goethite, ferrihydrite, kaolinite and montmorillonite by 14.0% (124.8 ± 2.7 to 107.3 ± 0.4), 55.5% (8.9 ± 0.2 to 3.9 ± 0.2), 41.1% (60.5 ± 0.6 to 35.6 ± 0.4) and 88.2% (155.4 ± 0.9 to 18.3 ± 7.6), respectively (Figure 1b). In contrast, hydroxyapatite showed no systematic trend and pH had a minimal effect on adsorption capacity, ranging from 12.8 ± 0.2 at pH 6.7 to 12.9 ± 0.3 at pH 8.9.

Electrostatic interactions play a key role in the pH-dependent adsorption of charged molecules, including DNA, with mineral surfaces.^12,24^ The isoelectric point of DNA is typically lower than 5,^67^ meaning gDNA is expected to carry a net negative charge across the entire experimental pH range. The point of zero charge (PZC) of goethite, ferrihydrite, and hydroxyapatite is 8.32, 7.9 and ∼7.6, respectively.^44,68^ In contrast, clays have a permanent negative layer charge (Table S3), with adsorption likely occurring at edge sites containing hydroxyl groups.^38,69,70^ The PZC at edge sites ranges from 5.6 to 6.6 for kaolinite and 9 to 10 for montmorillonite.^71^ Hence, all these minerals undergo a reversal of net surface charge (or edge charge for kaolinite) from positive to negative with increasing pH under our experimental conditions, except montmorillonite, which, despite a reduction in charge, maintains a net positive edge even at pH 9. Therefore, the decrease in adsorption on most minerals indicates decreasing electrostatic attraction between the negatively charged gDNA and the mineral surface with increasing pH until repulsive forces dominate their interaction. Despite this, adsorption occurs even at pH above the respective PZC of all minerals, demonstrating that adsorption is not purely driven by electrostatic interactions at alkaline pH.

As discussed above, the adsorption of gDNA to hydroxyapatite is relatively unaffected by charge reversal. We hypothesize that this behavior may be due to the presence of Ca^2+^ in the crystal lattice and in solution under solubility equilibrium with the mineral. Hydroxyapatite in our study has a hexagonal rod-like crystal structure carrying terminal {0001} (PZC 4.8) and Ca-rich lateral {101̅0} (PZC 8.7) surfaces,^72^ with an overall expected PZC ≥7 due to the dominance of lateral surfaces in rod-shaped crystals.^68^ While the overall hydroxyapatite surface charge varies with solution pH, gDNA adsorption may be driven by its binding to Ca-rich lateral surfaces, as polyelectrolytes could potentially navigate surfaces to find oppositely charged binding sites.^73^ Additionally, partial dissolution of hydroxyapatite, indicated by substantial drift from initial pH from 5 and 6 to around 6.8 (Figure 1b), would release Ca^2+^ ions in the submillimolar range (as predicted by PHREEQC equilibrium modeling, Figure S8), facilitating cation bridging across the pH range. The combination of binding to Ca-rich lateral surfaces and cation bridging may explain the minimal pH effect on DNA adsorption on hydroxyapatite.

### 3.3 Adsorption Isotherms of 99 bp and Genomic DNA

To investigate the binding affinity of DNA on mineral surfaces, we performed adsorption isotherms of gDNA and 99 bp DNA at initial concentrations ranging from 5–150 ng/μL at pH 7 for 6 h (Figure 1c–g). gDNA concentrations below the LOQ (<0.15 ng/μL) in suspension were observed at initial gDNA concentrations of ≤40 ng/μL for goethite, ≤10 ng/μL for ferrihydrite and hydroxyapatite and ≤25 ng/μL for kaolinite and montmorillonite, suggesting essentially complete adsorption. In case of 99 bp DNA, complete adsorption occurred at concentrations ≤40 ng/μL for goethite, ≤10 ng/μL for ferrihydrite and kaolinite, and ≤55 ng/μL for hydroxyapatite. The complete adsorption at low DNA loadings indicates high sorption affinities and sorbed concentrations below the maximum surface loading. As initial DNA concentrations increased in suspension, the maximum adsorption of DNA on the mineral surface was progressively approached and was eventually reached (except for gDNA on clays and hydroxyapatite), as evidenced by well-defined adsorption plateaux (Figure 1c–g). Interestingly, for montmorillonite, even at the lowest initial concentration of 5 ng/μL, complete adsorption of the 99 bp DNA from the solution was not achieved (Figure 1f). Moreover, montmorillonite exhibited a steady increase in adsorption with increasing gDNA concentrations and did not reach a plateau even at the highest initial concentration used in this experiment of 150 ng/μL, indicating a very high adsorption capacity for the longer polymer, consistent with previous studies.^22,74^

Complete DNA adsorption from suspension at low initial DNA concentrations implies a high affinity of 99 bp DNA and gDNA for these minerals (except for 99 bp on montmorillonite). To empirically quantify and compare the adsorption capacities, we fitted the adsorption data to the Langmuir and the Freundlich isotherm models (Tables S4 and S5). The maximum adsorption capacities derived from the Langmuir fit agreed well with our experimental data. However, it is important to note that these adsorption models do not always reflect the underlying mechanisms of polyelectrolyte adsorption on charged surfaces. The maximum gDNA adsorption capacities (in ng/cm^2^ from Langmuir fits, Figure 1h and Table S5) of the minerals used in this experiment were in the following order: montmorillonite > goethite > kaolinite > hydroxyapatite > ferrihydrite. For 99 bp DNA, adsorption capacities for minerals were different than the gDNA with the following order: goethite > kaolinite > hydroxyapatite ≈ montmorillonite > ferrihydrite. The maximum adsorption capacity of gDNA was significantly higher than that of 99 bp DNA on Fe­(III)-(oxyhydr)­oxides and clays, while hydroxyapatite showed the opposite trend (unpaired *t*-test, *p* < 0.05). To further investigate the role of polymer length in controlling DNA adsorption, we conducted additional experiments, as detailed in the following section.

### 3.4 Effect of DNA Polymer Length on Adsorption

To systematically investigate the effect of DNA polymer length on maximum adsorption capacity, we incubated 99 bp, 400 bp, 1000 bp, 4000 bp DNA, and gDNA (∼20,000 bp) at a uniform initial concentration of 20 ng/μL with minerals at pH 7 for 6 h. We modeled the maximum adsorption capacity as a function of DNA polymer length using an empirical power-law model as per eq 3, where *l* is the DNA polymer length (bp), and α is a scaling exponent (Table S6). While the power law model is frequently used to describe polymer adsorption on surfaces, it remains an empirical approach that does not account for site-specific interactions and polymer conformation during adsorption.^52,75^

3 $$\Gamma_{DNA}\propto l^{\alpha }$$

We observed an overall increase in maximum adsorption capacity (ng/cm^2^) with increasing polymer length for Fe­(III)-(oxyhydr)­oxides and clays, but a decrease for hydroxyapatite (Figure 2a–e), consistent with the trends observed in the adsorption isotherms. Specifically, adsorption (ng/cm^2^) of gDNA relative to 99 bp DNA on goethite, ferrihydrite, kaolinite and montmorillonite increased by 88.5% (69.0 ± 1.4 to 130.1 ± 1.8; α = 0.12), 58.8% (3.4 ± 0.2 to 5.4 ± 0.1; α = 0.10), 86.0% (22.1 ± 1.5 to 41.1 ± 3.5; α = 0.10), and 753% (22.0 ± 7.3 to 187.6 ± 1.1; α = 0.36), respectively, and decreased by 39.2% (20.6 ± 0.3 to 12.5 ± 0.2; α = −0.12) on hydroxyapatite. This trend is further reflected in the value of scaling exponent α, where a positive value for Fe­(III)-(oxyhydr)­oxides and clays indicates increasing coverage adsorption with polymer length and vice versa for hydroxyapatite. Finally, the estimated surface area occupied by adsorbed DNA polymers at maximum adsorption capacity was less than the total available mineral surface area (except for gDNA on montmorillonite), suggesting that not all surface sites were occupied (Figure S12), indicating most likely less than monolayer adsorption for most minerals in our experiments. However, in the case of montmorillonite, considering adsorption on edge sites, a possibility of locally exceeding monolayer adsorption cannot be completely ruled out.

**Figure 2:**
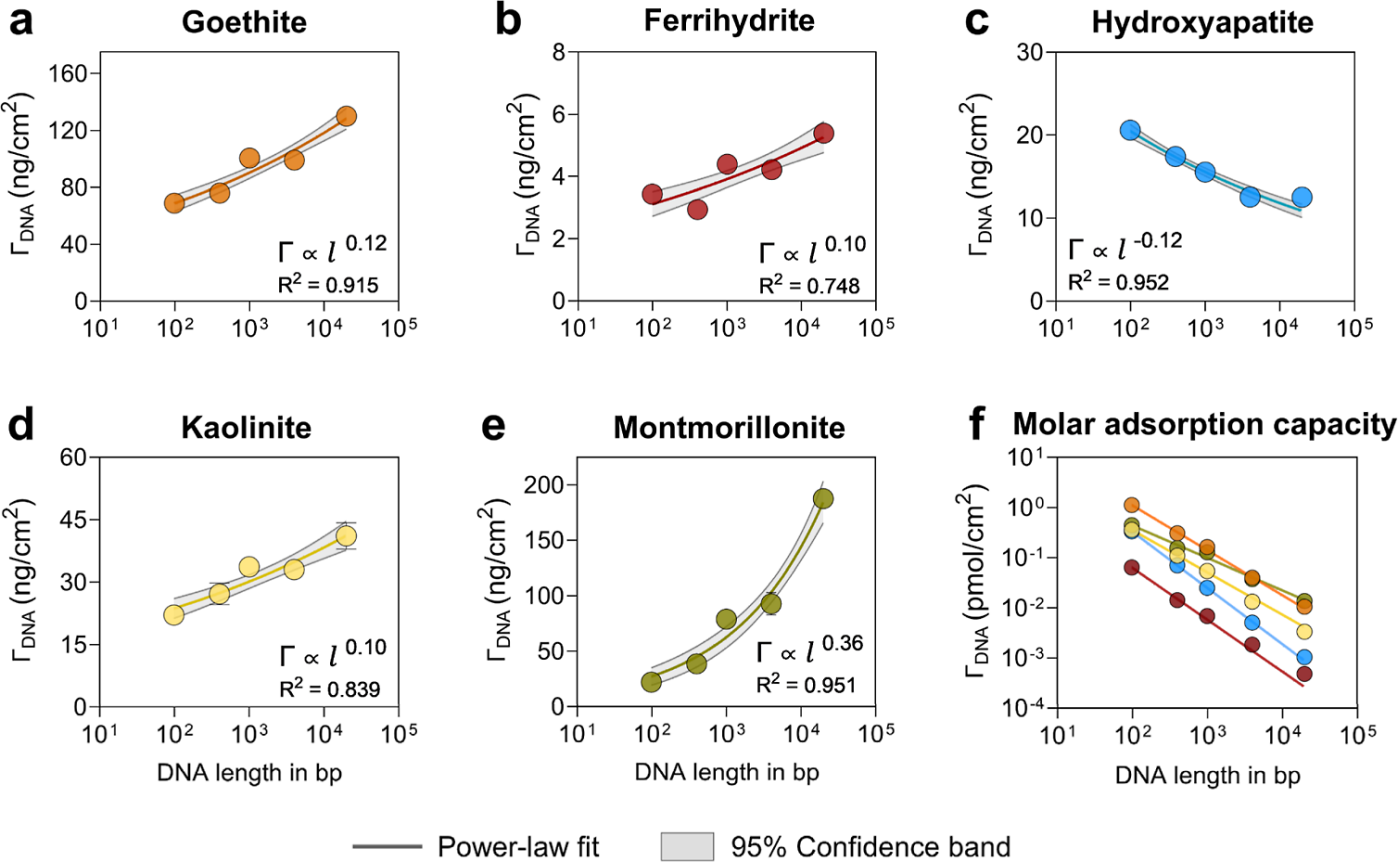
Influence of DNA polymer length on maximum adsorption capacity. 20 ng/μL of 99, 400, 1000, 4000 bp and gDNA (∼20,000 bp) were incubated separately with minerals at pH 7 for 6 h in a solution containing 3 mM HEPES and 30 mM NaCl. High initial DNA concentrations and lower mineral loadings (Section 2.3.2) ensured complete surface coverage; therefore, each data point represents the maximum adsorption capacity of the respective DNA polymer on each mineral. Data points and error bars represent the mean and standard deviation of triplicates run in parallel.

To explain the influence of polymer length on DNA adsorption capacity, the bending properties of DNA polymers, their dimensions compared to mineral particles, charge reversal upon adsorption and binding to specific surface sites must be considered. For instance, 99 bp, 400 bp, 1000 bp, 4000 bp DNA, and gDNA (∼20,000 bp) used in this study have an estimated maximum end-to-end length of 0.0337, 0.136, 0.340, 1.36 and ∼6.8 μm, assuming a 0.34 nm base stacking distance in B-form DNA.^76^ DNA behaves as a stiff rod below its persistence length, typically around 50 nm or 150 bp, due to repulsion between negatively charged backbone phosphate groups.^77^ Charged mineral surfaces can further induce DNA bending by screening its charge and raising local ionic strength, though shorter polymers are still expected to bend comparatively less than longer ones.^78^ Therefore, in our experiments, 99 bp likely behaves as a rigid rod, while longer polymers up to gDNA likely bend and coil progressively with increasing polymer length. These differences in DNA flexibility, combined with polymer dimensions exceeding those of mineral particles or particle aggregates, may shape DNA–mineral conjugate architecture and influence adsorption capacity.

Previous studies show that long polyelectrolytes, including DNA polymers, adsorbed onto charged surfaces can adopt a “loop–train–tail” configuration, where “train” refers to sections of the polymers bound to the surface while “loops” and “tails” extend away from the respective attachment points.^39,66,69,79–81^ Furthermore, DNA adsorption, irrespective of polymer length, imparts a more negative charge (charge overcompensation) across all minerals, except on montmorillonite, as reflected by reduced zeta potential values (Figure S7). For short, flat-lying DNA polymers, this negative charge leads to electrostatic repulsion between DNA–mineral conjugates, resulting in well-dispersed suspensions (*e.g.*, goethite, Figure S9). In contrast, long polymers, despite the charge overcompensation, can extend beyond the electrical double layer and, owing to their higher flexibility and multiple binding sites, can bridge multiple mineral particles, inducing flocculation^48,82–84^ (*e.g.*, goethite, Figure S9) or gel formation (*e.g.*, montmorillonite).^69^ We hypothesize that the close surface association of short polymers increases their surface footprint—occupying more surface area per molecule—and, together with charge overcompensation, inhibits the approach of DNA from solution toward the surface, collectively contributing to lower DNA mass adsorption. In contrast, loop–train–tail configuration by long polymers reduces surface footprint per molecule, and, together with bridging-induced flocculation and gel formation, may collectively contribute to the higher adsorption plateau. This mechanism potentially explains the increasing adsorption capacity with increasing polymer length for Fe­(III)-(oxyhydr)­oxides and clays. However, it is important to note that the polymer organization on mineral surfaces, mineral particle aggregation, and charge overcompensation that govern the length-dependent adsorption trend are highly sensitive to factors controlling the electrostatic environment, *e.g.*, pH, ionic strength, and ion valency.^52,64,69^

Finally, adsorption can also be modulated by binding to structurally or functionally distinct surface sites, as revealed by previously reported spectroscopic and imaging studies.^22,54,56,57,62^ For example, adsorption to clays primarily occurs at the edge sites exposing hydroxyl groups.^22,54,59,81^ At pH 7, the edge sites of montmorillonite carry an overall positive charge (PZC_edge_ = 9–10), while kaolinite carries a negative charge (PZC_edge_ = 5.6–6.6).^71^ Flexible DNA polymers can navigate across surfaces to locate positively charged sites,^65^ likely leading to greater gDNA adsorption on montmorillonite compared to kaolinite, despite montmorillonite having a higher overall negative surface charge. In contrast to clays, Fe­(III)-(oxyhydr)­oxides provide the same Fe–OH groups across the crystal faces, leading to likely nonselective binding. However, the variable surface density and spatial distribution of these Fe–OH groups across crystallographic faces may affect adsorption.^85^ Similarly, adsorption to hydroxyapatite crystals, which have a hexagonal rodlike shape, likely occurs on lateral surfaces with exposed Ca atoms as discussed before.^69,70^

Total adsorption onto ferrihydrite was unexpectedly low across all polymer lengths in our experiments. While ferrihydrite typically exhibits high adsorption of several inorganic species, including phosphate, adsorption of organic species varies widely depending on molecular size, functional groups, and mineral particle aggregation.^86,87^ For example, when expressed in terms of phosphate group adsorption, all DNA polymers adsorb 2 orders of magnitude lower phosphate (10^–8^ mol/m^2^) than inorganic phosphate (10^–6^ mol/m^2^),^86^ on ferrihydrite, under comparable solution conditions. Given that DNA binds primarily through phosphate groups, this disparity is unlikely due to the weak binding affinity of DNA. Instead, we attribute the reduced adsorption to nanoparticle aggregation in our suspension (also potentially resulting from the freeze-drying step during synthesis), limiting the available surface area, consistent with lower adsorption for other organic compounds on such freeze-dried ferrihydrite.^88^

SEM images of hydroxyapatite reveal aggregates up to 200 μm (Figure S10), despite prior ultrasonication, which is well-documented for hydroxyapatite synthesized by chemical precipitation methods.^89,90^ However, adsorption on <2 μm hydroxyapatite fraction showed a similar trend, suggesting that large aggregates likely did not contribute to the observed trend (Figure S10). Higher adsorption of short polymers on hydroxyapatite likely arises from its highly porous nature, reflected by high pore volume (0.7 cm^3^/g) and large pore diameter (ranging up to 60 nm) (Figure S3),^91^ which may induce size exclusion of longer polymers while allowing preferential incorporation of smaller ones into pore spaces. A similar size exclusion phenomenon leading to higher adsorption of short polymers has been observed in model soils.^37^ Despite varying mass adsorption, the molar adsorption of DNA consistently decreased for all minerals as the polymer length increased (Figure 2f). This is because longer polymers have higher molar mass, leading to fewer molecules adsorbed overall, despite their higher mass adsorption on Fe­(III)-(oxyhydr)­oxides and clays.

### 3.5 Competitive Adsorption of DNA with Varying Polymer Lengths

In natural environments, coexisting DNA of varying polymer lengths may compete for surface binding sites, potentially influencing DNA fate through preferential binding or displacement. We investigated these processes by performing competitive adsorption experiments under simultaneous and sequential addition scenarios (Figures 3, S13.1–S13.5), using 99 bp DNA, representing a highly degraded/fragmented DNA (utilized in eDNA and aDNA studies) and 2000 bp DNA, representing partially degraded nuclear or plasmid DNA. DNA loadings of each polymer (15 ng/μL) aimed for complete surface saturation, so that the adsorption of the subsequently added polymer could only occur through competitive displacement of the previously adsorbed polymer.

**Figure 3:**
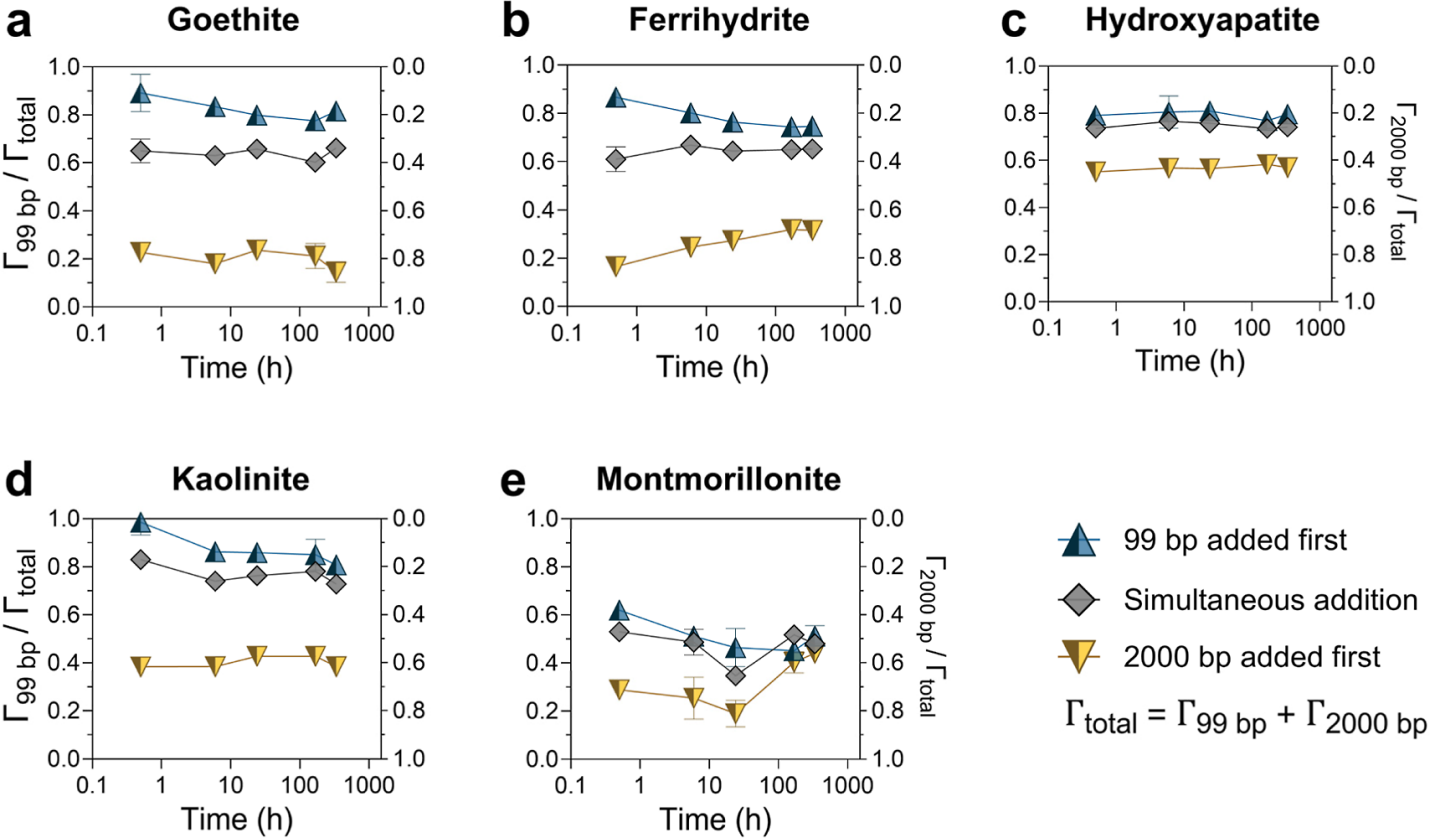
Relative adsorption of 99 and 2000 bp DNA (mass normalized) under competitive conditions. A total of three competitive adsorption experiments were conducted. (i) Both 99 and 2000 bp DNA were added simultaneously to the suspension with minerals. (ii) Minerals were first incubated with 99 bp DNA for 30 m, followed by the addition of 2000 bp DNA. (iii) Minerals were first incubated with 2000 bp DNA for 30 m, followed by the addition of 99 bp DNA. The final concentrations of both the DNA polymers were 15 ng/μL each, sufficient to reach adsorption plateaus for each polymer individually. The background solution contained 3 mM HEPES and 30 mM NaCl at pH 7. Data points and error bars represent the mean and standard deviation of triplicates run in parallel.

Under simultaneous addition, 99 bp DNA exhibited higher adsorption than 2000 bp DNA across all minerals except montmorillonite. This can be attributed to the diffusion-limited initial adsorption step, favoring faster adsorption of shorter polymers from nonuniform suspensions, reflected by 8.7 times higher diffusion coefficient of 99 bp DNA over 2000 bp DNA (Table S2).^64,92,93^ For instance, during the adsorption of single DNA polymers on goethite under control conditions (Figure S13.1), 79.5% of the 99 bp DNA was adsorbed within just 30 min relative to its 24 h adsorption, compared to only 67.9% for the 2000 bp DNA—indicating a faster adsorption rate for shorter polymers. At the two-week time point, the mass fraction of 99 bp adsorbed was 0.661 ± 0.013 on goethite, 0.652 ± 0.007 on ferrihydrite, 0.727 ± 0.025 on kaolinite and 0.741 ± 0.036 on hydroxyapatite. In contrast, montmorillonite showed a mass fraction of 0.480 ± 0.016 for 99 bp adsorption, highlighting no significant size-specific preference for either of the polymers. During sequential addition, the order of addition was determinative and irrespective of polymer length for goethite, ferrihydrite and kaolinite, with the first introduced DNA displaying greater adsorption. However, for montmorillonite, the first introduced polymer, whether it was 99 or 2000 bp, showed higher adsorption initially but converged to similar levels to those seen during simultaneous addition by 2 weeks. For hydroxyapatite, the relative adsorption of 99 bp DNA was consistently higher, even when 2000 bp was added first.

The adsorption trends across three competitive scenarios did not converge within 2 weeks, except for montmorillonite, highlighting a kinetically limited, nonequilibrium state. Interestingly, the absolute adsorption amount of both 99 bp and 2000 bp DNA did not decrease under any competitive conditions (Figures S13.1–S13.5), indicating that the observed relative changes in adsorption were not driven by competitive displacement. Instead, the later-added polymer increasingly adsorbed over time, while the amount of initially adsorbed polymers remained relatively unchanged, thereby reducing its relative proportion. This is surprising, considering that the high initial DNA loadings should have been sufficient to achieve maximum or near-maximum surface saturation (considering a slow increase in adsorption over time, Figure 1a), meaning the adsorption of later-added polymer should, in principle, have occurred via competitive displacement of initially adsorbed polymer. Nevertheless, the increased adsorption of the later-added polymer, without displacement of the initial polymer, may be due to binding to the sites that became accessible through the steric reorganization of the first adsorbed polymer.

4 $${\Delta G}_{DNA\;adsorption}={\Delta H}_{bond\;formation}-T\left({\Delta S}_{counter\;ion\;release}+{\Delta S}_{configurational\;restrictions\;upon\;adsorption}+{\Delta S}_{configurational\;flexibility\;in\;solution}\right)$$

The adsorption of the polymer (99 bp vs 2000 bp DNA), resulting in the most negative Gibbs free energy, considering enthalpic and entropic contributions (eq 3), will be thermodynamically favored in a nonuniform system. Inner-sphere complexation and electrostatic attraction (at pH 7) with positively charged surfaces of goethite, ferrihydrite, and hydroxyapatite, and edges of montmorillonite contribute to negative enthalpy, while repulsion from negatively charged kaolinite surface contributes to positive enthalpy. Repulsion between charged segments within the same polymer or adjacent polymers (coadsorbed at the surface or lying above the surface-bound polymer) further contributes to a positive enthalpy. These different binding mechanisms vary in their energetic contributions. Despite these varying enthalpic contributions, the overall polyelectrolyte adsorption on charged surfaces is primarily driven by entropy.^64,92,94,95^ The entropic gain associated with counterion liberation at coverage adsorption can be considered similar for both polymers, as nearly all binding sites are occupied, leading to the displacement of an equal number of counterions. The entropic loss (penalty) associated with configurational restriction upon adsorption is expected to be higher for longer polymers, favoring the adsorption of shorter ones. However, there is typically far greater entropic gain by having more short polymers in suspension, which can acquire more configurational states than fewer, longer polymer with the equivalent total mass, implying longer polymers should preferentially adsorb at thermodynamic equilibrium.^64,92,94^ Therefore, even if the initial adsorption step favors the adsorption of 99 bp DNA, during simultaneous addition and when 99 bp was added first in our experiments, they should in principle be displaced by 2000 bp over time. In contrast, higher adsorption of shorter polymers observed here further highlights a kinetically limited, nonequilibrium state.

Kinetically favored adsorption of shorter 99 bp DNA during simultaneous addition leads to charge overcompensation on positively charged minerals (e.g., Fe­(III)-(oxyhydr)­oxides, and hydroxyapatite) or even stronger negative charge on negatively charged kaolinite, due to a high negative charge density at DNA backbone, as reflected from zeta potential measurements (Figure S7). A similar charge overcompensation is expected to occur by coverage adsorption of the first introduced polymer during sequential addition experiments. This negative charge creates an electrostatic barrier preventing DNA in solutions from approaching the surface and displacing the previously adsorbed polymers, thereby slowing down or inhibiting the equilibration. Furthermore, displacement requires simultaneous breaking of multiple attachment points of adsorbed DNA, which is statistically less likely on shorter time scales and therefore a slow process. A similar kinetically limited state with higher adsorption of shorter polymers during competitive adsorption has been observed for other polyelectrolytes on hematite,^94,96^ barium sulfate,^97,98^ and other charged surfaces.^64,99^

### 3.6 Environmental Implications

This study offers critical mechanistic insights into the role and significance of DNA polymer length for DNA adsorption to mineral surfaces. Our findings reveal a rapid initial DNA adsorption to all tested minerals, which may contribute to the swift immobilization of DNA and, therefore, protection from degradation, particularly against enzymatic hydrolysis in soil and sediments.^13–16^ Our results have direct significant implications for the fate and persistence of DNA for eDNA and aDNA applications. For instance, based on our data, we can infer that goethite-rich soils (*e.g.*, oxisols, ultisols, *etc.*) may serve as potential archives for short DNA polymers relevant to aDNA recovery, whereas, both smectite- (*e.g.*, vertisols) and goethite-rich soils could act as reservoirs for longer DNA polymers, which are useful for eDNA analysis. High adsorption of short DNA polymers on hydroxyapatite further highlights its well-recognized potential for exceptional preservation of aDNA in bones^32^ as evident by bone fragments being highly sought after as aDNA source in field samplings. However, in natural environments, the presence of organic matter^13^ and inorganic species (*e.g.*, phosphate)^24^ may reduce DNA adsorption by competition and occupying surface binding sites and therefore, should be considered accordingly while sampling. Furthermore, due to the high adsorption capacity of long DNA polymers, goethite and montmorillonite can be suitable materials for constructing cost-effective passive eDNA samplers for eDNA sampling from aquatic environments.

We hypothesized that extracellular DNA degradation generates a nonuniform DNA pool, where polymers of varying lengths compete on the surface for adsorption sites. Our competitive adsorption experiments reveal consistent preferential adsorption of shorter (99 bp) polymers over longer (2000 bp) ones up to 2 weeks, suggesting a kinetically controlled process, as the adsorption of long polymers should be favored thermodynamically. Given the rapid decay of DNA in aquatic environments with half-lives up to 1.5 weeks^1^ at circumneutral pH, nonadsorbed longer DNA polymers would likely degrade further before having the opportunity to replace shorter polymers through adsorption. This mechanism may provide insights into the relative preservation of different DNA polymer lengths in environmental systems, particularly the exclusive preservation of short DNA fragments (<100 bp) over long-term periods.^32^ Our findings underscore the need for further mechanistic studies addressing the effect of DNA polymer length while studying environmental degradation and preservation.

The favored preservation of short DNA polymers has important implications in diverse applications. In the context of aDNA, the stability of short DNA polymers (<100 bp) on mineral surfaces may be key to their long-term survival and retrievability from environmental samples. However, in biodiversity monitoring, this persistence could be problematic, as qPCR-based species detection, which often targets short eDNA amplicons, may result in false positives, i.e., detection of species that are no longer present.^100,101^ Additionally, the prevalence and persistence of short DNA polymers suggest that mineral-adsorbed short DNA may constitute a significant global phosphorus reservoir.^9^ Furthermore, these small DNA polymers adsorbed on mineral surfaces may contribute to bacterial evolution and antibiotic resistance^19^ over extended time scales, as natural transformation can occur by highly degraded DNA as small as 20 bp.^33^

## Supplementary Material



## Notes

DNA synthesis and purification; mineral preparation and characterization; DNA quantification method from a nonuniform solution; DNA stability under experimental conditions; Freundlich, Langmuir and power-law model fitting of DNA adsorption data; changes in surface zeta potential upon DNA adsorption; additional adsorption experiments; extended adsorption data (PDF)
