# Supplementary material for: Polymer Length Governs DNA Adsorption Dynamics on Mineral Surfaces
Source: Environ Sci Technol. 2025 Sep 18;59(38):20462–73. doi: 10.1021/acs.est.5c08180 (PMC12489982; doi:10.1021/acs.est.5c08180)
Supplement: Supplementary file 1 [file es5c08180_si_001.pdf]

Supporting Information for

Polymer length governs DNA adsorption dynamics on mineral surfaces

Veer Vikram Singh[1,2], Naresh Kumar[3], Richard L. Kimber[4*], Ákos Weiser[1], Ron Pinhasi[5], Stephan M. Kraemer[1]*

[1]*Department of Environmental Geosciences, University of Vienna, Josef-Holaubek-Platz 2, 1090, Vienna, Austria*

[2]*Doctoral School in Microbiology and Environmental Science, University of Vienna, Josef-Holaubek-Platz 2, 1090, Vienna, Austria*

[3]*Soil Chemistry, Wageningen University and Research, Droevendaalsesteeg 3, 6708 PB Wageningen, the Netherlands*

[4]*Department of Earth and Environmental Sciences, The University of Manchester, Manchester, M13 9PL, UK*

[5]*Department of Evolutionary Anthropology, University of Vienna, Djerassiplatz 1, 1030 Vienna, Austria*

*Corresponding author: naresh.kumar@wur.nl

richard.kimber@manchester.ac.uk

Number of pages: 32

Number of figures: 17

Number of tables: 6

Table of Contents

Section S1: Materials **S3**

Table S1: Details of chemicals and kits S3

Section S2: Details of DNA polymers **S4**

S2.1 PCR synthesis, purification, and quality assessment S4
S2.2: Sequence of plasmid pBR322 and amplified DNA polymers S5
Table S2: Details of primers and DNA polymers used in the study S6
S2.3: DNA polymer length determination using automated gel electrophoresis S7
Figure S1: Size distribution of DNA polymers S7

S3: Mineral synthesis and characterization **S8**

S3.1: Iron oxide synthesis S8
S3.2: Preparation of homoionic clays S8
S3.3: Hydroxyapatite synthesis S8
S3.4: Mineral characterization S9
S3.4.1: Mineralogy S9
S3.4.2: Specific surface area and pore dimensions S9
Figure S2: X-ray diffractograms of minerals S10
Table S3: Physicochemical properties of minerals S11
Figure S3: Porous structural data of selected minerals S12

Section S4: Methods **S13**

Figure S4: Quantification of individual DNA polymer from a non-uniform solution S13
Figure S5: Solution phase stability of gDNA S15
Figure S6: Solution phase stability of 99 and 2000 bp DNA in a mixed system S16

Section S5: Model fitting of DNA adsorption data **S17**

Table S4: Model fit parameters for the non-linear Freundlich adsorption isotherm model S18
Table S5: Model fit parameters for the non-linear Langmuir adsorption isotherm model S19
Table S6: Power-law fit parameters S20

Section S6: Additional adsorption experiments **S21**

Figure S7: Changes in zeta potential values of mineral surface upon DNA adsorption S21
Figure S8: Effect of dissolved Ca^{2+} on DNA adsorption S22
Figure S9: Flocculation of goethite upon DNA adsorption S23
Figure S10: Effect of hydroxyapatite particle size on adsorption S24

Section S7: Extended adsorption data **S25**

Figure S11: Detailed adsorption isotherms S25
Figure S12: Relative surface coverage of adsorbed DNA on minerals S26
Figure S13.1: Competitive adsorption on Goethite S27
Figure S13.2: Competitive adsorption on Ferrihydrite S28
Figure S13.3: Competitive adsorption on Kaolinite S29
Figure S13.4: Competitive adsorption on Montmorillonite S30
Figure S13.5: Competitive adsorption on Hydroxyapatite S31

References **S32**

Section S1: Materials

Vendor	*Chemical/kits*
Merck	Sodium acetate trihydrate (certified ACS)
	Sodium tetraborate decahydrate (≥99.5%, certified ACS)
	Sodium chloride (certified ACS)
	Potassium hydroxide (for analysis)
	Iron (III) nitrate nonahydrate (99.0-101.0 %, certified ACS),
	0.1 μm Protein low-binding PVDF syringe filter
	Ethanol absolute GR for analysis (≥99.9%, certified ACS)
	Isopropanol absolute GR for analysis (≥99.9%, certified ACS)
Sigma-Aldrich	DNA sodium salt from Salmon testes
Thermo Fisher Scientific	Hydrochloric acid (certified ACS Plus)
	Ammonia solution (28%)
	pBR322 plasmid DNA (0.5 μg/μL)
	DreamTaq DNA polymerase (5U/μL)
	Qubit 1X dsDNA broad-range assay kits (including buffer, standards, and assay tubes)
Carl ROTH	4-(2-hydroxyethyl)-1-piperazineethanesulfonic acid (HEPES, ≥ 99.5%, for biochemistry and molecular biology)
	2-(N-morpholino)ethanesulfonic acid (MES, ≥ 99.5%, for biochemistry and molecular biology)
	Tris-(hydroxymethyl)-aminomethane hydrochloride (Tris-HCl, for molecular biology)
	Dialysis membrane Spectra/Por® 7 MWCO 3500
Microsynth Austria	Desalted primers for PCR (100 μM)
Qiagen	MinElute PCR Purification Kit
Agilent Technologies	D1000, D5000 and genomic DNA screen tapes
	Reagents (buffer & ladders) for D1000, D5000 and genomic DNA screen tapes
Amresco	Sodium hydroxide (reagent grade)
Eppendorf	Protein LoBind tubes (2 ml, nuclease-free)
Biozym	Pipette tips (nuclease-free)

Table S1: Details of chemicals and kits

Section S2: Details of DNA polymers

S2.1 PCR synthesis, purification, and quality assessment

We used polymerase chain reaction (PCR) to synthesize 99, 400, 1000, 2000 and 4000 bp DNA polymers. For each 50 μL reaction mixture, we mixed 42.25 μL of ultrapure water, 5 μL of 10X DreamTaq buffer, 1 μL of dNTP mix (10 mM of each nucleotide), 0.5 μL of forward primer (100 μM), 0.5 μL of reverse primer (100 μM), 0.5 μL of plasmid pBR322 as template (2 ng/μL) and finally 0.25 μL of DreamTaq DNA polymerase (5 U/μL) in a 200 μL PCR tube. All reagents were kept on ice during mixing to prevent any nonspecific amplification. The thermal cycler parameters included an initial denaturation (95 °C, 1.5 min), followed by 35 cycles of denaturation (95 °C, 30 s), primer annealing (56 °C, 30 s) and extension (72 °C, 1 min for 99–2000 bp and 3 min for 4000 bp), followed by a final extension step at 72 °C for 5 min. The sequence of the plasmid template and the sequence and location of PCR products are provided in section S2.2. PCR-synthesized DNA polymers were purified using MinElute PCR Purification Kit (Qiagen, USA) as per the manufacturer's instructions. After purification, the DNA was eluted into the desired 2X reaction buffers (i.e., 6 mM buffer species, 60 mM NaCl), as required for the experiments. We prepared the genomic DNA (gDNA) solution by directly dissolving sodium salts provided by the manufacturer in the desired 2X buffers for 3h. The absorbance ratio A_{260}/A_{280} for all DNA solutions was between 1.8–1.9, ensuring no protein contamination, while A_{260}/A_{230} ratio ranged between 2.0–2.2, ensuring no carryover of chaotropic salts from PCR cleanup.

S2.2: Sequence of plasmid pBR322 and amplified DNA polymers

5’–TTCTCATGTTTGACAGCTTATCATCGATAAGCTTTAATGCGGTAGTTTATCACAGTTAAATTGCTAACGCAGTCAGGCACCGTGTATGAAATCTA
ACAATGCGCTCATCGTCATCCTCGGCACCGTCACCCTGGATGCTGTAGGCATAGGCTTGGTTATGCCGGTACTGCCGGGCCTCTTGCGGGATATCG
TCCATTCCGACAGCATCGCCAGTCACTATGGCGTGCTGCTAGCGCTATATGCGTTGATGCAATTTCTATGCGCACCCGTTCTCGGAGCACTGTCCG
ACCGCTTTGGCCGCCGCCCAGTCCTGCTCGCTTCGCTACTTGGAGCCACTATCGACTACGCGATCATGGCGACCACACCCGTCCTGTGGATCCTCTA
CGCCGGACGCATCGTGGCCGGCATCACCGGCGCCACAGGTGCGGTTGCTGGCGCCTATATCGCCGACATCACCGATGGGGAAGATCGGGCTCGC
CACTTCGGGCTCATGAGCGCTTGTTTCGGCGTGGGTATGGTGGCAGGCCCCGTGGCCGGGGGACTGTTGGGCGCCATCTCCTTGCATGCACCATT
CCTTGCGGCGGCGGTGCTCAACGGCCTCAACCTACTACTGGGCTGCTTCCTAATGCAGGAGTCGCATAAGGGAGAGCGTCGACCGATGCCCTTGA
GAGCCTTCAACCCAGTCAGCTCCTTCCGGTGGGCGCGGGGCATGACTATCGTCGCCGCACTTATGACTGTCTTCTTTATCATGCAACTCGTAGGAC
AGGTGCCGGCAGCGCTCTGGGTCATTTTCGGCGAGGACCGCTTTCGCTGGAGCGCGACGATGATCGGCCTGTCGCTTGCGGTATTCGGAATCTTG
CACGCCCTCGCTCAAGCCTTCGTCACTGGTCCCGCCACCAAACGTTTCGGCGAGAAGCAGGCCATTATCGCCGGCATGGCGGCCGACGCGCTGGG
CTACGTCTTGCTGGCGTTCGCGACGCGAGGCTGGATGGCCTTCCCCATTATGATTCTTCTCGCTTCCGGCGGCATCGGGATGCCCGCGTTGCAGGC
CATGCTGTCCAGGCAGGTAGATGACGACCATCAGGGACAGCTTCAAGGATCGCTCGCGGCTCTTACCAGCCTAACTTCGATCACTGGACCGCTGA
TCGTCACGGCGATTTATGCCGCCTCGGCGAGCACATGGAACGGGTTGGCATGGATTGTAGGCGCCGCCCTATACCTTGTCTGCCTCCCCGCGTTG
CGTCGCGGTGCATGGAGCCGGGCCACCTCGACCTGAATGGAAGCCGGCGGCACCTCGCTAACGGATTCACCACTCCAAGAATTGGAGCCAATCA
ATTCTTGCGGAGAACTGTGAATGCGCAAACCAACCCTTGGCAGAACATATCCATCGCGTCCGCCATCTCCAGCAGCCGCACGCGGCGCATCTCGG
GCAGCGTTGGGTCCTGGCCACGGGTGCGCATGATCGTGCTCCTGTCGTTGAGGACCCGGCTAGGCTGGCGGGGTTGCCTTACTGGTTAGCAGAA
TGAATCACCGATACGCGAGCGAACGTGAAGCGACTGCTGCTGCAAAACGTCTGCGACCTGAGCAACAACATGAATGGTCTTCGGTTTCCGTGTTT
CGTAAAGTCTGGAAACGCGGAAGTCAGCGCCCTGCACCATTATGTTCCGGATCTGCATCGCAGGATGCTGCTGGCTACCCTGTGGAACACCTACA
TCTGTATTAACGAAGCGCTGGCATTGACCCTGAGTGATTTTTCTCTGGTCCCGCCGCATCCATACCGCCAGTTGTTTACCCTCACAACGTTCCAGTA
ACCGGGCATGTTCATCATCAGTAACCCGTATCGTGAGCATCCTCTCTCGTTTCATCGGTATCATTACCCCCATGAACAGAAATCCCCCTTACACGGA
GGCATCAGTGACCAAACAGGAAAAAACCGCCCTTAACATGGCCCGCTTTATCAGAAGCCAGACATTAACGCTTCTGGAGAAACTCAACGAGCTGG
ACGCGGATGAACAGGCAGACATCTGTGAATCGCTTCACGACCACGCTGATGAGCTTTACCGCAGCTGCCTCGCGCGTTTCGGTGATGACGGTGAA
AACCTCTGACACATGCAGCTCCCGGAGACGGTCACAGCTTGTCTGTAAGCGGATGCCGGGAGCAGACAAGCCCGTCAGGGCGCGTCAGCGGGTG
TTGGCGGGTGTCGGGGCGCAGCCATGACCCAGTCACGTAGCGATAGCGGAGTGTATACTGGCTTAACTATGCGGCATCAGAGCAGATTGTACTG
AGAGTGCACCATATGCGGTGTGAAATACCGCACAGATGCGTAAGGAGAAAATACCGCATCAGGCGCTCTTCCGCTTCCTCGCTCACTGACTCGCT
GCGCTCGGTCGTTCGGCTGCGGCGAGCGGTATCAGCTCACTCAAAGGCGGTAATACGGTTATCCACAGAATCAGGGGATAACGCAGGAAAGAAC
ATGTGAGCAAAAGGCCAGCAAAAGGCCAGGAACCGTAAAAAGGCCGCGTTGCTGGCGTTTTTCCATAGGCTCCGCCCCCCTGACGAGCATCACA
AAAATCGACGCTCAAGTCAGAGGTGGCGAAACCCGACAGGACTATAAAGATACCAGGCGTTTCCCCCTGGAAGCTCCCTCGTGCGCTCTCCTGTT
CCGACCCTGCCGCTTACCGGATACCTGTCCGCCTTTCTCCCTTCGGGAAGCGTGGCGCTTTCTCATAGCTCACGCTGTAGGTATCTCAGTTCGGTGT
AGGTCGTTCGCTCCAAGCTGGGCTGTGTGCACGAACCCCCCGTTCAGCCCGACCGCTGCGCCTTATCCGGTAACTATCGTCTTGAGTCCAACCCGG
TAAGACACGACTTATCGCCACTGGCAGCAGCCACTGGTAACAGGATTAGCAGAGCGAGGTATGTAGGCGGTGCTACAGAGTTCTTGAAGTGGTG
GCCTAACTACGGCTACACTAGAAGGACAGTATTTGGTATCTGCGCTCTGCTGAAGCCAGTTACCTTCGGAAAAAGAGTTGGTAGCTCTTGATCCG
GCAAACAAACCACCGCTGGTAGCGGTGGTTTTTTTGTTTGCAAGCAGCAGATTACGCGCAGAAAAAAAGGATCTCAAGAAGATCCTTTGATCTTTT
CTACGGGGTCTGACGCTCAGTGGAACGAAAACTCACGTTAAGGGATTTTGGTCATGAGATTATCAAAAAGGATCTTCACCTAGATCCTTTTAAATT
AAAAATGAAGTTTTAAATCAATCTAAAGTATATATGAGTAAACTTGGTCTGACAGTTACCAATGCTTAATCAGTGAGGCACCTATCTCAGCGATCT
GTCTATTTCGTTCATCCATAGTTGCCTGACTCCCCGTCGTGTAGATAACTACGATACGGGAGGGCTTACCATCTGGCCCCAGTGCTGCAATGATACC
GCGAGACCCACGCTCACCGGCTCCAGATTTATCAGCAATAAACCAGCCAGCCGGAAGGGCCGAGCGCAGAAGTGGTCCTGCAACTTTATCCGCCT
CCATCCAGTCTATTAATTGTTGCCGGGAAGCTAGAGTAAGTAGTTCGCCAGTTAATAGTTTGCGCAACGTTGTTGCCATTGCTGCAGGCATCGTGG
TGTCACGCTCGTCGTTTGGTATGGCTTCATTCAGCTCCGGTTCCCAACGATCAAGGCGAGTTACATGATCCCCCATGTTGTGCAAAAAAGCGGTTA
GCTCCTTCGGTCCTCCGATCGTTGTCAGAAGTAAGTTGGCCGCAGTGTTATCACTCATGGTTATGGCAGCACTGCATAATTCTCTTACTGTCATGCC
ATCCGTAAGATGCTTTTCTGTGACTGGTGAGTACTCAACCAAGTCATTCTGAGAATAGTGTATGCGGCGACCGAGTTGCTCTTGCCCGGCGTCAAC
ACGGGATAATACCGCGCCACATAGCAGAACTTTAAAAGTGCTCATCATTGGAAAACGTTCTTCGGGGCGAAAACTCTCAAGGATCTTACCGCTGTT
GAGATCCAGTTCGATGTAACCCACTCGTGCACCCAACTGATCTTCAGCATCTTTTACTTTCACCAGCGTTTCTGGGTGAGCAAAAACAGGAAGGCA
AAATGCCGCAAAAAAGGGAATAAGGGCGACACGGAAATGTTGAATACTCATACTCTTCCTTTTTCAATATTATTGAAGCATTTATCAGGGTTATTG
TCTCATGAGCGGATACATATTTGAATGTATTTAGAAAAATAAACAAATAGGGGTTCCGCGCACATTTCCCCGAAAAGTGCCACCTGACGTCTAAGA
AACCATTATTATCATGACATTAACCTATAAAAATAGGCGTATCACGAGGCCCTTTCGTCTTCAAGAA–**3’**

Colored sequence in plasmid template

99 bp	1000 bp	4000 bp	(a) 20 bp	Forward primer for DNA (start location)
400 bp	2000 bp		(b) 1 bp	Endpoint for DNA sequence

Length (bp)	*Length (μm)*[a]	*Molar weight ($\times 10^6$ Da)*	*Diffusion coefficient ($\times 10^{-8}$ cm²/s)*[b]	*Nucleobase composition of DNA (%)*					*Primer sequence (5'–3') used in DNA synthesis*
				A	*T*	*G*	*C*	*G+C*	
99	0.034	0.06	17.92	26.3	16.2	24.2	33.3	57.6	Forward: TACGGGAGGGCTTACCATCT<br>Reverse: TTCCGGCTGGCTGGTTTATT
400	0.136	0.25	6.56	24.0	19.0	26.0	31.0	57.0	Forward: CGCACAGATGCGTAAGGAGA<br>Reverse: CTTCCCGAAGGGAGAAAGGC
1000	0.34	0.62	3.39	23.9	20.7	26.3	29.1	55.4	Forward: ACCGCCAGTTGTTTACCCTC<br>Reverse: TTGGAGCGAACGACCTACAC
2000	0.68	1.24	2.06	21.4	21.1	28.1	29.5	57.6	Forward: CGGCCTCAACCTACTACTGG<br>Reverse: CCACCTCTGACTTGAGCGTC
4000	1.36	2.47	1.25	21.9	23.1	26.8	28.3	55.0	Forward: GCATAGGCTTGGTTATGCCG<br>Reverse: AACATTTCCGTGTCGCCCTT
gDNA	~6.8	~12.3	0.39	–	–	–	–	–	–

Table S2: Details of primers and DNA polymers used in the study.

[a]Length of linear DNA assuming B-form in solution with 0.34 nm distance between base pairs. [1]

[b]Expected diffusion coefficients of linear DNA in ultrapure water, estimated using an empirical formula from Ref. 2.

S2.3: DNA polymer length determination using automated gel electrophoresis

DNA polymer lengths were estimated using an automated gel electrophoresis system, Agilent Tapestation 4150, following the manufacturer's instructions using pre-assembled ScreenTapes analogous to conventional agarose gels. Briefly, DNA samples were mixed with the fluorescent dye-containing buffer and loaded into the instrument. The instrument runs the mixture on the ScreenTapes under an electric field, forcing DNA to migrate toward the positive electrode through the gel. Due to the porous nature of gels, short DNA polymers travel further than the longer polymers, giving a size distribution of DNA in gel, which is visualized by measuring the fluorescence of DNA along the gel length. The polymer length of DNA is then assigned by comparing it against the standard DNA ladder.

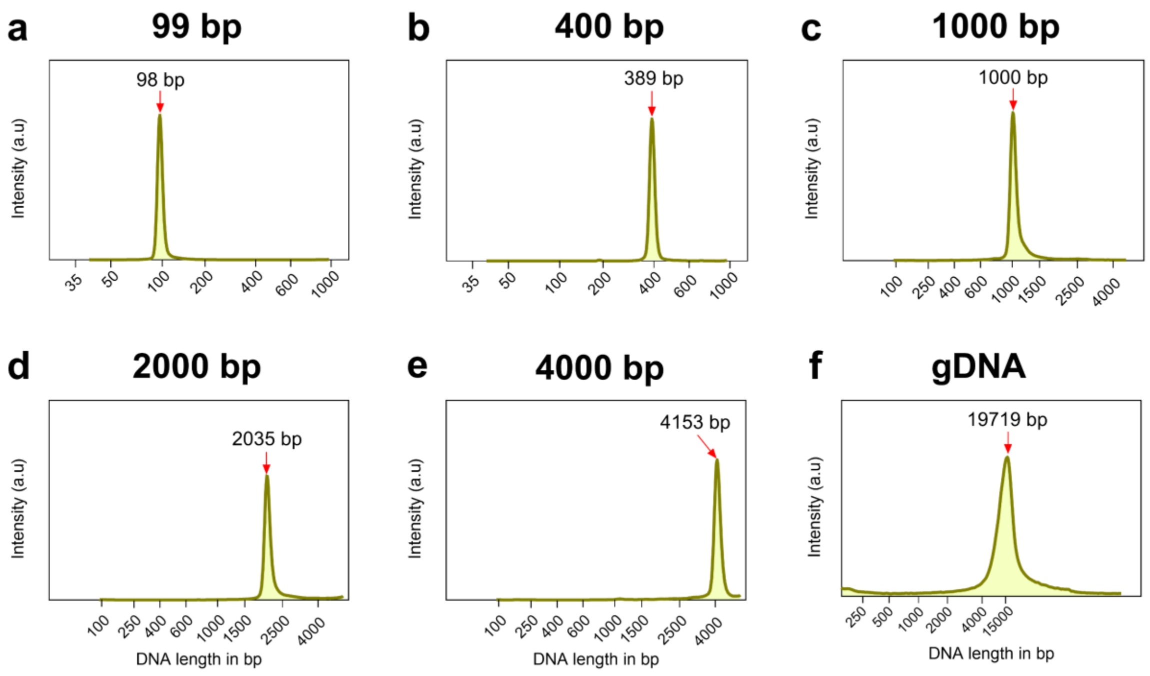

Figure S1: Size distribution of DNA polymers. 20 ng/μL DNA in 3 mM HEPES, 30 mM NaCl, pH 7, measured using an automated gel electrophoresis system. **(a)** 99 bp and **(b)** 400 bp DNA polymers were measured on D1000 ScreenTape, **(c)** 1000 bp, **(d)** 2000 bp, and **(e)** 4000 bp DNA polymers using D5000 ScreenTape, and **(f)** gDNA was measured on genomic DNA ScreenTape. The measured median DNA polymer length is indicated by a red arrow in the figures, which lies well within the sizing accuracy specified by the manufacturer: ± 10% for D1000 and D5000 and ± 15% for Genomic DNA ScreenTapes. For clarity, raw fluorescence intensity data of samples were re-plotted after aligning the internal standard peaks in the sample images against the internal standard peaks of raw data using GraphPad Prism (version 10.4.1). Internal standards were excluded afterwards from the plots for simplicity.

S3: Mineral synthesis and characterization

S3.1: Iron oxide synthesis

The Fe(III)-(oxyhydr)oxides were synthesized using the alkaline precipitation method from Schwertmann and Cornell 1991.[3] For 2-line ferrihydrite synthesis, 500 mL of 0.1 M $Fe(NO_3)_3.9H_2O$ was mixed with 150 mL of 1 M NaOH under vigorous stirring. The pH of the mixture was immediately adjusted to 7.5 by dropwise addition of 1 M NaOH. For goethite synthesis, 100 ml of 1M $Fe(NO_3)_3.9H_2O$ was mixed rapidly with 180 ml of 5M KOH under vigorous stirring in a polyethylene flask, resulting in precipitation of ferrihydrite. The resulting suspension was immediately diluted to 2 liters and incubated at 70 °C in an oven for 60 h, transforming ferrihydrite into goethite. The precipitated iron oxides were washed six times with ultrapure water to remove excess ions.

S3.2: Preparation of homoionic clays

The homoionic clays were prepared using the protocol of Groeningen, N.V. et al., 2021.[4] 15 g of clays were dispersed separately in 1 litre ultrapure water (specific resistance >18.2 MΩ cm), followed by ultrasonication at 35 kHz for 5 minutes (Bandelin Sonorex RK 100, Bandelin electronics, Germany) and thorough mixing on a magnetic stirrer (RH basic 2, IKA Werke, Germany) at 200 rpm for an hour for better dispersion of clay particles. The suspension was centrifuged at 100 g for 5 minutes, and the supernatant with clay particles <2 μm hydrodynamic diameter was separated. This process was repeated until the suspension was free from clay particles. The suspension was then saturated with Na^+ by adding an appropriate amount of NaCl to achieve a concentration of 1M and mixed on a magnetic stirrer at 300 rpm for 24 h. The suspension was then centrifuged at 7000 g for 1 h. To ensure complete Na^+ saturation, the saturation step was repeated two more times by adding 1 M NaCl solution. After cation saturation, the excess salt was removed by dialyzing the clay suspension (Dialysis membrane Spectra/Por® 7 MWCO 3500) until the conductivity reached <2 μS/cm.

S3.3: Hydroxyapatite synthesis

Biomimetic hydroxyapatite was synthesized using the chemical precipitation method modified from Li et al., 2019.[5] Briefly, 400 ml of 0.2 M $Ca(NO_3)_2{\cdot}4H_2O$ and 240 ml of 0.2 M $(NH_4)_2HPO_4$ were prepared separately. The pH of the $(NH_4)_2HPO_4$ solution was adjusted to 11 by adding ammonia (NH_3) solution. The $Ca(NO_3)_2{\cdot}4H_2O$ solution was then added dropwise into the $(NH4)_2HPO_4$ solution under vigorous stirring. The pH during the mixing was kept above 11 and set to 11.5 by adding NH_3 once the mixing was done. For optimal crystal growth, the mixture was incubated at 30 °C for 24 h, followed by six washes with ultrapure water (30 minutes at 7000 g) to remove any excess ions.

After dialysis or washing, all minerals were freeze-dried, grounded, and stored in amber glass tubes either at 4 °C (for goethite, kaolinite, and montmorillonite) or at −80 °C (ferrihydrite and hydroxyapatite) to minimize mineral transformation.

S3.4: Mineral characterization

S3.4.1: Mineralogy

Mineralogical characterization was performed by measuring powder X-ray diffraction (XRD) spectra of minerals on a Rigaku MiniFlex diffractometer (Rigaku, Miniflex 600), using Cu-Kα radiation (λ = 1.5406 Å). Mineral identification was performed using Profex 5.2.1.[6] We matched the diffraction peaks of the samples with reference structure files (BGMN) from software and found that goethite, kaolinite, and hydroxyapatite samples are pure and do not contain any other mineralogical phases. Ferrihydrite showed two broad peaks, confirming the presence of a 2-line ferrihydrite. The diffractogram of the <2 μm fraction of montmorillonite reveals minor quartz and feldspar impurities, consistent with observations reported in baseline studies of source clays by the Clay Minerals Society and other recent research.[7,8] Despite the minor presence of quartz, montmorillonite is still the major constituent (>95% by weight) and is expected to contribute foremost surface reactivity owing to its high specific surface area compared to quartz.[7]

S3.4.2: Specific surface area and pore dimensions

The specific surface area of the minerals was measured on Quantachrome Nova 2000e using the six-point Brunauer-Emmett-Teller (BET) method. The pore volumes and mean pore diameters of selected minerals were measured using the Barrett-Joyner-Halenda (BJH) method. The minerals were degassed overnight at room temperature before the N_2 adsorption.

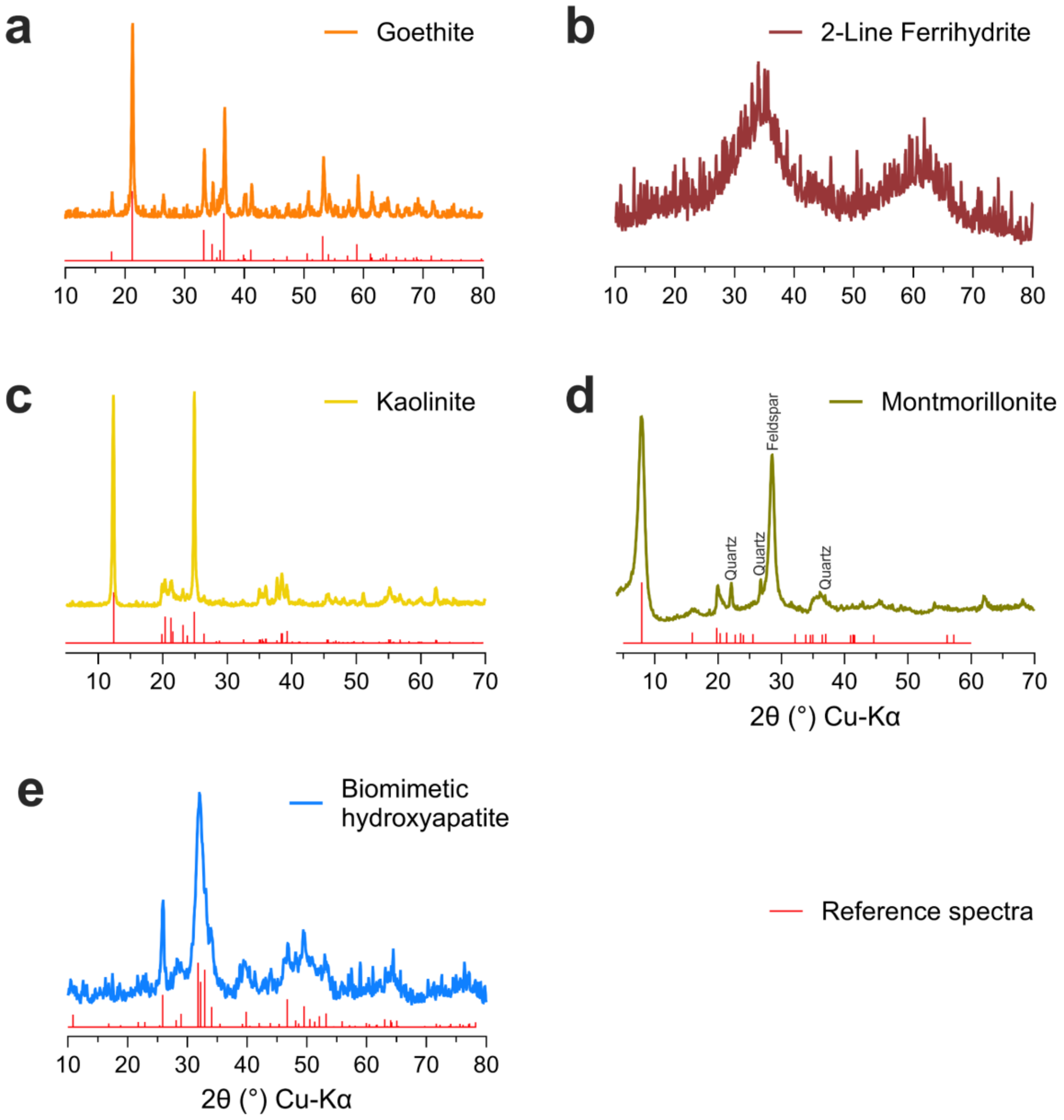

Figure S2: X-ray diffractograms of minerals. Diffraction spectra of mineral powders were recorded between 5-70 °2θ for clays and between 10-80 °2θ for the rest of the minerals at 0.05° step size and 0.5°/min scanning speed on a zero-background silica sample holder.

Mineral	SSA[a] ($m^2 g^{-1}$)	PZC[b]	CEC[c] (meq/100 g)	Layer charge[9]	Longest primary particle dimension
Goethite	30.2 ± 1.5	~7.6[10]	–	–	0.5 – 5 μm[d]
Ferrihydrite	244.7 ± 12.7	~7.9[10]	–	–	~5 nm[10]
Kaolinite (KGa-1b)	16.7 ± 1.3	5.9 ± 0.1[11]	3.0 ± 0.1[12]	Octahedral charge: 0.11<br>Tetrahedral charge: -0.17<br>Interlayer charge: -0.06<br>Unbalanced charge: 0.00	< 2 μm[e]
Montmorillonite (SWy-3)	49.8 ± 1.5	9.6 ± 0.0[11]	85 ± 3[12]	Octahedral charge: -0.53<br>Tetrahedral charge: -0.02<br>Interlayer charge: -0.55<br>Unbalanced charge: 0.05	< 2 μm[e]
Hydroxyapatite	222.8 ± 9.6	7.6 ± 0.1[13]	–	–	<100 nm[d] (Fig S10)

Table S3: Physicochemical properties of minerals

[a]Specific Surface Area (mean from triplicate measurements ± standard deviation) as determined by N_2-BET in the current study.
[b]Point of zero charge. pH at which the surface of Fe(III)-(oxyhydr)oxides and edges of clays have a net zero charge.
[c]Cation exchange capacity.
[d]Particle dimensions measured using TEM in the current study.
[e]Particle dimension cutoff used during gravitational settling in the current study.

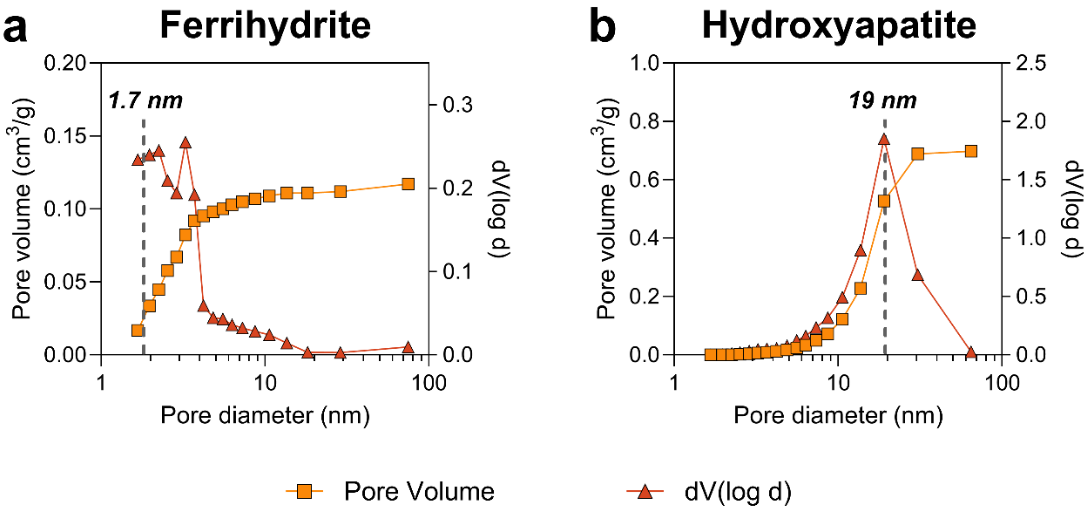

Figure S3: Porous structural data of selected minerals. Mean pore diameters estimated using the Barrett-Joyner-Halenda (BJH) method are indicated in the figure.

Section S4: Methods

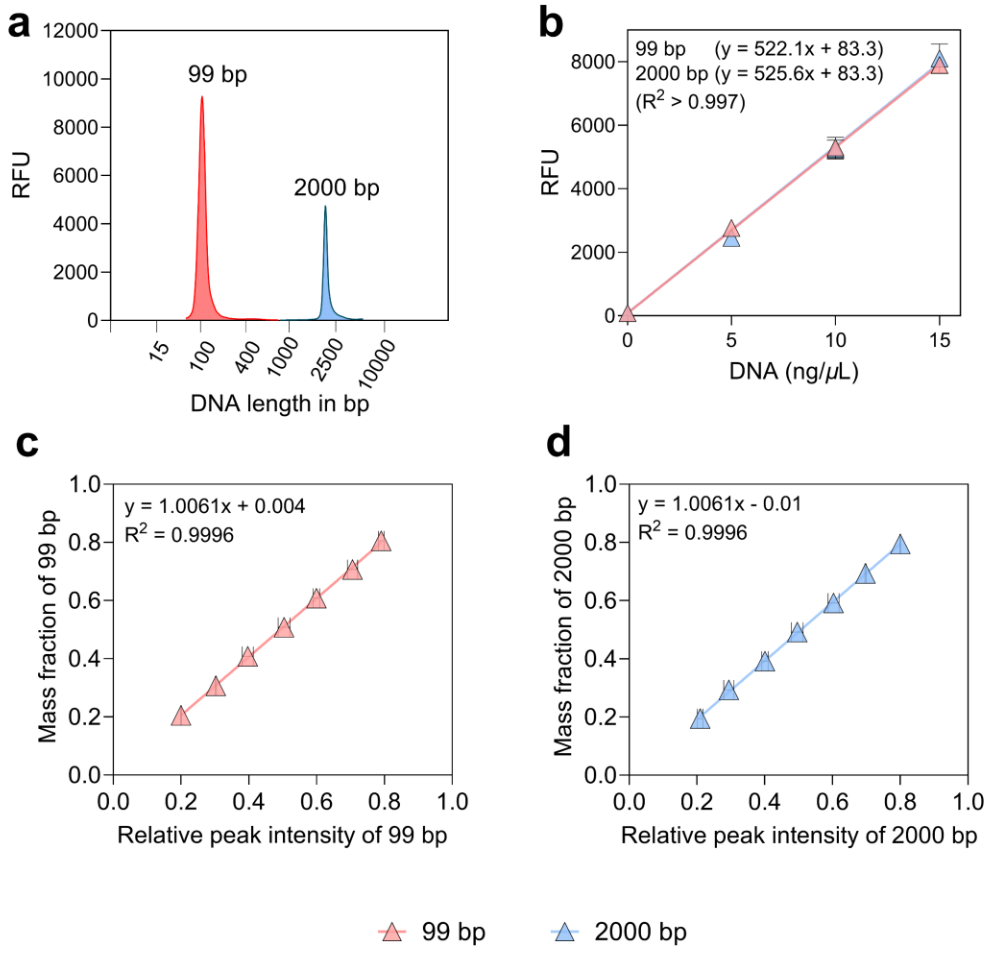

Figure S4: Quantification of individual DNA polymer from a non-uniform solution. We observed that the mass fractions of 99 or 2000 bp DNA from a non-uniform, bi-dispersed solution correlated proportionally to their relative peak intensities measured on D5000 ScreenTape. Therefore, we utilized this information to construct a calibration curve for quantifying the concentrations of individual DNA polymers in an unknown bi-dispersed experimental solution from the competitive adsorption experiments. We prepared standard solutions with varying mass ratios of 99 and 2000 bp DNA polymers, measured their relative peak intensities on D5000 ScreenTape, and performed linear regression to construct calibration curves. Finally, the absolute concentrations of each DNA polymer were determined by multiplying their relative mass fractions by the total DNA concentration, using equations (1) and (2). **(a)** An example of an electropherogram (using D5000 ScreenTape) with a 70:30 mass ratio of 99 and 2000 bp DNA. Internal markers have been removed for clarity. **(b)** Fluorometric intensities of 99 bp and 2000 bp DNA in a uniform solution show similar fluorescence, implying that in a non-uniform bimodal system (as in competitive adsorption experiments), the fluorescence of individual polymer is proportional to its mass fraction. **(c & d)** Calibration curves of 99 and 2000 bp DNA with varying mass fractions, up to a total concentration of 15 ng/μL and their corresponding

relative peak intensities with fitted linear regression. Data points and error bars represent the mean and standard deviation of triplicates run in parallel.

$$DNA_{99\,bp} = DNA_{total} \times \left(\frac{RFU_{99\,bp}}{RFU_{99\,bp} + RFU_{2000\,bp}} \times 1.0061 + 0.004 \right) \quad (1)$$

$$DNA_{2000\,bp} = DNA_{total} \times \left(\frac{RFU_{2000\,bp}}{RFU_{99\,bp} + RFU_{2000\,bp}} \times 1.0061 - 0.01 \right) \quad (2)$$

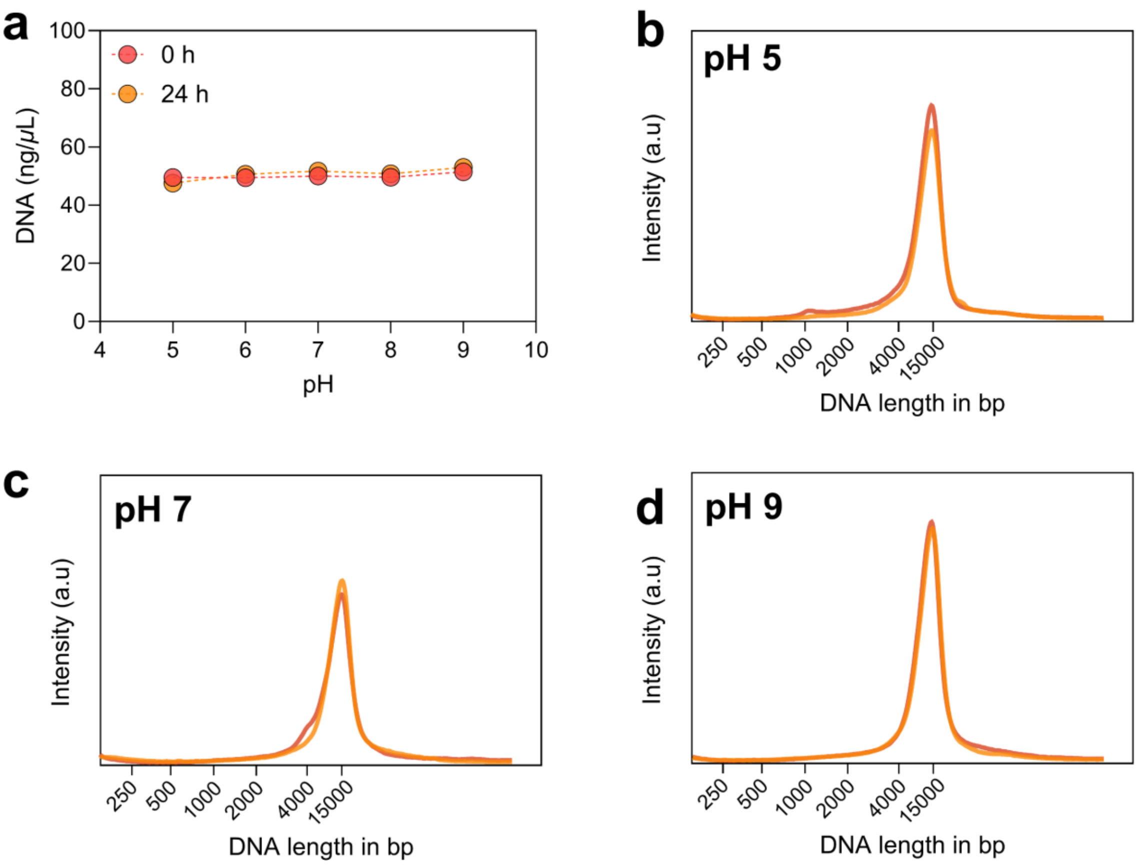

Figure S5: Solution phase stability of gDNA. Solutions with 50 ng/μL gDNA in 30 mM NaCl and 3 mM buffer (sodium acetate for pH 5, MOPS for pH 6, HEPES for pH 7 and 8, and sodium tetraborate for pH 9) were incubated at 20 °C for 24 h, centrifuged (20,000 rcf, 40 min) and subsequently analyzed for **(a)** concentration and **(b-d)** size distribution on gDNA ScreenTapes. The internal markers have been removed from the electropherograms for clarity. Symbols and error bars in (a) represent the mean and range of concentrations, whereas lines in (b-d) represent the mean fluorescent intensity from duplicates run in parallel. These results demonstrate that there was no detectable loss of gDNA due to biotic or abiotic degradation, or sedimentation during centrifugation.

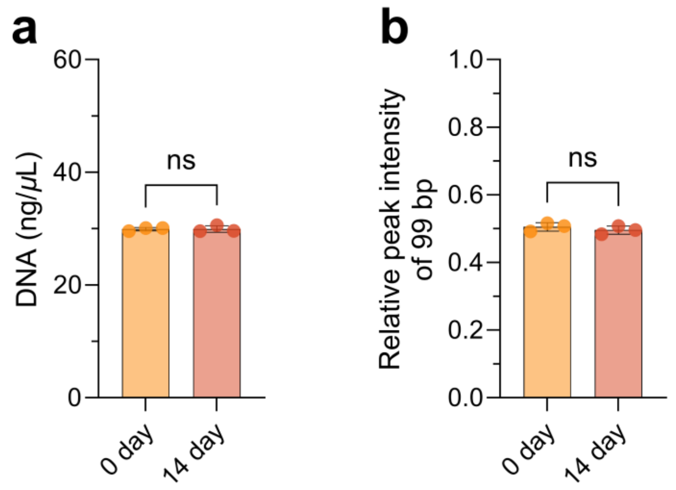

Figure S6: Solution phase stability of 99 and 2000 bp DNA in a mixed system. Solutions containing both DNA polymers (15 ng/μL each) in 30 mM NaCl and 3 mM HEPES at pH 7 were incubated at 20 °C for 14 days, centrifuged (20,000 rcf, 40 min), and subsequently analyzed for **(a)** concentration and **(b)** relative peak intensity of each DNA polymer. Symbols represent individual data points; bar height and error bars represent mean and standard deviations of triplicates run in parallel. There was no significant variation in DNA concentrations and relative peak intensities for up to two weeks (paired t-test, $p > 0.05$), highlighting no DNA loss due to decay or centrifugation-driven sedimentation.

Section S5: Model fitting of DNA adsorption data

Adsorption isotherm data were fitted with a non-linear form of the Freundlich (equation 3) and Langmuir adsorption models (equation 4).

$$\Gamma = K_f (C_e)^{1/n} \tag{3}$$

$$\Gamma = \frac{\Gamma_{max}\, k_L\, C_e}{1 + k_L C_e} \tag{4}$$

Here, K_f denotes the Freundlich constant, C_e denotes the DNA concentration (ng/μL) left in the solution at equilibrium, n denotes the Freundlich intensity parameter, Γ_{max} represent the maximum adsorption capacity, and k_L denotes the Langmuir constant.

Minerals	***99 bp***						***Genomic DNA***					
	$K_{f,area}$	n_{area}	R^2	$K_{f,mass}$	n_{mass}	R^2	$K_{f,area}$	n_{area}	R^2	$K_{f,mass}$	n_{mass}	R^2
Goethite	57.7 ± 0.2	10.5 ± 0.2	0.55 ± 0.01	17.4 ± 0.1	10.5 ± 0.2	0.55 ± 0.02	100.0 ± 1.4	13.7 ± 0.4	0.69 ± 0.01	30.2 ± 0.4	13.7 ± 0.4	0.68 ± 0.01
Ferrihydrite	2.5 ± 0.2	18.8 ± 8.8	-0.21 ± 0.13	6.3 ± 0.5	22.4 ± 14.0	-0.20 ± 0.13	3.5 ± 0.2	12.9 ± 3.6	0.69 ± 0.06	8.6 ± 0.6	13.5 ± 3.8	0.68 ± 0.06
Kaolinite	24.5 ± 0.1	72.4 ± 54.9	0.20 ± 0.09	4.1 ± 0.0	85.2 ± 78.8	0.19 ± 0.09	37.6 ± 0.6	11.8 ± 1.6	0.89 ± 0.02	6.3 ± 0.1	11.8 ± 1.6	0.66 ± 0.42
Montmorillonite	3.2 ± 1.3	3.1 ± 1.4	0.20 ± 0.24	2.3 ± 1.8	9.2 ± 8.5	0.11 ± 0.31	37.0 ± 1.5	2.4 ± 0.0	0.90 ± 0.01	18.4 ± 0.8	2.4 ± 0.0	0.9 ± 0.01
Hydroxyapatite	15.8 ± 1.0	7.8 ± 0.8	0.70 ± 0.04	35.1 ± 2.2	7.8 ± 0.8	0.71 ± 0.04	3.4 ± 0.5	4.3 ± 0.4	0.82 ± 0.03	7.2 ± 1.1	4.2 ± 0.4	0.82 ± 0.03

Table S4: Model fit parameters for the non-linear Freundlich adsorption isotherm model investigated in this study for 99 bp DNA and gDNA adsorption onto minerals.

Units

$$K_{f,area} = \frac{ng/cm^2}{(ng/\mu L)^{1/n}}$$

$$K_{f,mass} = \frac{\mu g/mg}{(ng/\mu L)^{1/n}}$$

$$n = dimensionless$$

Minerals	***99 bp***						***Genomic DNA***					
	$\Gamma_{max,area}$	$k_{L,area}$	R^2	$\Gamma_{max,mass}$	$k_{L,mass}$	R^2	$\Gamma_{max,area}$	$k_{L,area}$	R^2	$\Gamma_{max,mass}$	$k_{L,mass}$	R^2
Goethite	78.6 ± 0.7	28.4 ± 0.9	0.70 ± 0.01	23.7 ± 0.2	28.4 ± 0.9	0.70 ± 0.01	127.0 ± 3.5	7.5 ± 0.5	0.68 ± 0.01	38.3 ± 1.0	7.5 ± 0.4	0.68 ± 0.01
Ferrihydrite	3.3 ± 0.1	0.5 ± 0.2	-0.21 ± 0.14	8.2 ± 0.4	0.6 ± 0.3	-0.20 ± 0.14	4.9 ± 0.4	0.8 ± 0.2	0.68 ± 0.06	11.9 ± 1.1	0.8 ± 0.2	0.68 ± 0.06
Kaolinite	26.9 ± 1.1	1.6 ± 0.5	0.20 ± 0.09	4.5 ± 0.2	1.6 ± 0.6	0.20 ± 0.09	51.3 ± 3.1	6.1 ± 0.9	0.86 ± 0.01	8.6 ± 0.5	6.1 ± 0.9	0.62 ± 0.42
Montmorillonite	18.0 ± 8.5	0.2 ± 0.2	0.18 ± 0.23	4.6 ± 1.2	0.3 ± 0.3	0.12 ± 0.28	222.2 ± 2.7	0.1 ± 0.0	0.87 ± 0.02	110.6 ± 1.3	0.1 ± 0.0	0.87 ± 0.02
Hydroxyapatite	22.1 ± 0.7	3.3 ± 0.2	0.70 ± 0.04	49.2 ± 1.6	3.3 ± 0.2	0.70 ± 0.04	9.7 ± 0.6	0.2 ± 0.0	0.74 ± 0.05	21.0 ± 1.3	0.2 ± 0.0	0.74 ± 0.05

Table S5: Model fit parameters for the non-linear Langmuir adsorption isotherm model investigated in this study for 99 bp DNA and gDNA adsorption onto minerals.

Units

$\Gamma_{max,area} = ng/cm^2$

$\Gamma_{max,mass} = \mu g/mg$

$k_L = (ng/\mu L)^{-1}$

Mineral	$\Gamma_{DNA}\left(\frac{ng}{cm^2}\right) \propto l^{\alpha}$		$\Gamma_{DNA}\left(\frac{pmol}{cm^2}\right) \propto l^{\alpha}$	
	α	R^2	α	R^2
Goethite	0.117	0.915	-0.894	0.999
Ferrihydrite	0.099	0.748	-1.030	0.990
Kaolinite	0.104	0.839	-0.842	0.992
Montmorillonite	0.361	0.951	-0.312	0.382
Hydroxyapatite	-0.119	0.952	-1.120	1.000

Table S6: Power-law fit parameters for the maximum DNA adsorption capacity as a function of polymer length l (in bp) (Figure 2, main text). A positive value of α indicates increasing adsorption capacity with polymer length, while a negative value indicates a decreasing trend.

Section S6: Additional adsorption experiments

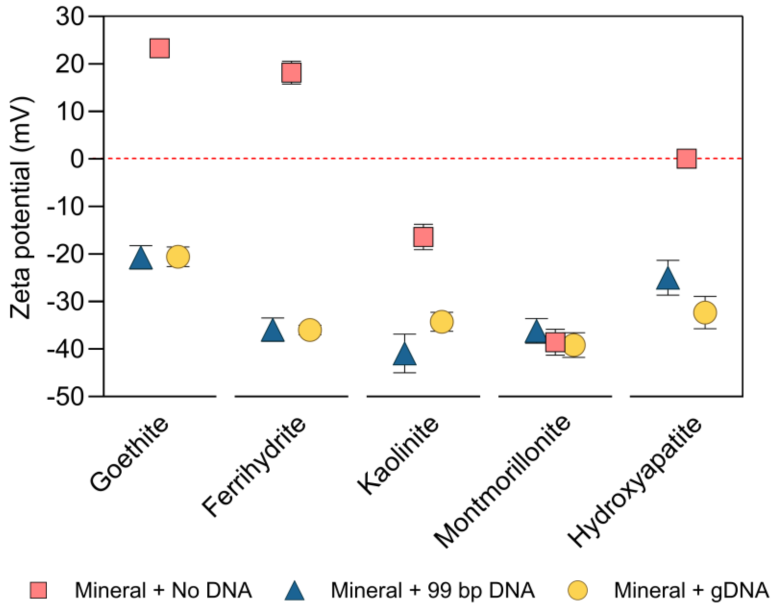

Figure S7: Changes in zeta potential values of mineral surface upon DNA adsorption. Minerals were incubated with either 2.5 ng/μL of 99 bp DNA or genomic DNA (~20,000 bp) at pH 7 for 6 hours in a solution containing 3 mM HEPES and 30 mM NaCl. The low DNA concentration ensured complete adsorption onto the mineral surfaces. Data points represent the mean of duplicate experiments run in parallel, whereas error bars denote the propagated uncertainty calculated from the standard deviations of individual replicates.

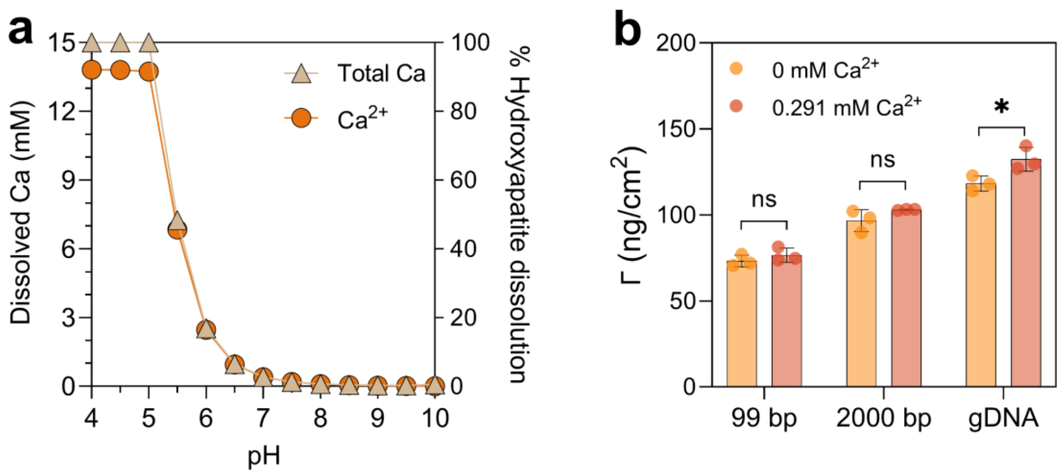

Figure S8: Effect of dissolved Ca^{2+} on DNA adsorption. **(a)** Predicted Ca^{2+} release into solution from hydroxyapatite dissolution during the pH-dependent DNA adsorption experiment (Figure 1b, main text), calculated using PHREEQC equilibrium modeling with the PHREEQC database. **(b)** We hypothesized that if the dissolved Ca^{2+} (0.291 mM) during hydroxyapatite adsorption experiments contributed to the higher adsorption of shorter DNA polymers compared to longer ones (Figures 1g and 2c, main text), it should also enhance the adsorption of shorter polymers onto goethite. Therefore, to test this hypothesis, we incubated 20 ng/μL of each 99 bp DNA, 2000 bp DNA and gDNA with 0.3 mg/ml of goethite in the background of 3 mM HEPES, 30 mM NaCl, at pH 7, with 0 or 0.291 mM $CaCl_2$ for 6 h. The results indicate that the adsorption on goethite increased with increasing DNA polymer length (consistent with Figures 1c and 2a, main text), and dissolved Ca^{2+} does not enhance the adsorption of shorter DNA polymers. The presence of 0.291 mM Ca^{2+} had no significant effect on the adsorption of 99 and 2000 bp DNA ($p > 0.05$) but significantly increased gDNA adsorption ($p =$ 0.0154) compared to no Ca^{2+} controls (unpaired t-test). Bar heights and error bars represent the mean and standard deviation of triplicates run in parallel.

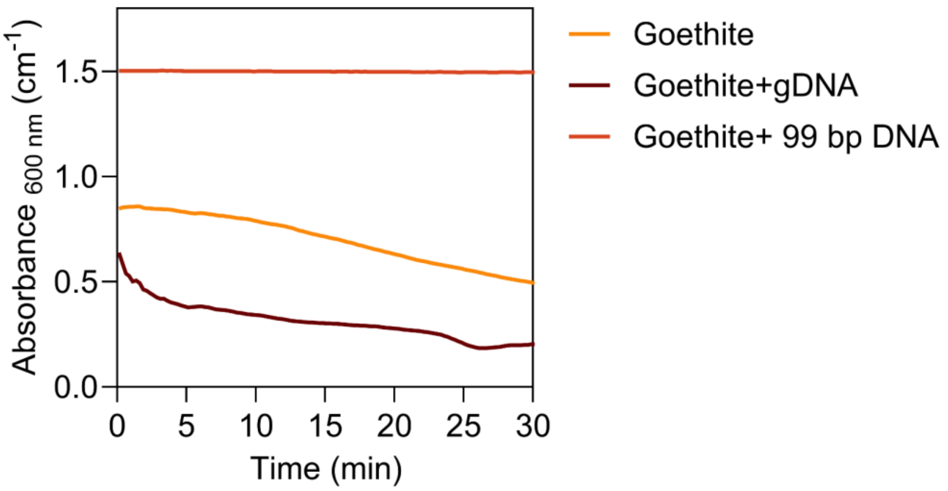

Figure S9: Flocculation of goethite upon DNA adsorption. 20 ng/μL of 99 bp DNA and gDNA were mixed with 0.2 mg/ml goethite up to the final volume of 1 ml and incubated for 6 hours. Afterwards, the solution was transferred into quartz cuvettes and loaded into a UV-visible spectrophotometer and absorbance at 600 nm was measured for up to 30 minutes. A decrease in absorbance indicates sedimentation of goethite or goethite-DNA conjugates. In the control solution containing only goethite, slow sedimentation was observed over time. In contrast, the goethite adsorbed with 99 bp DNA showed no sedimentation, due to charge overcompensation upon adsorption (Figure 3, main text), which repelled the goethite-99 bp conjugates and maintained a well-dispersed suspension. However, the goethite adsorbed with gDNA settled very rapidly due to flocculation, despite the similar extent of charge overcompensation as of goethite 99 bp-conjugates, suggesting that the long gDNA (~6.8 μm) extends beyond the electric double layer and bridges multiple goethite particles. The data points on the line represent the mean from the duplicate scans, and error bars, representing the range, were within the dimensions of the lines.

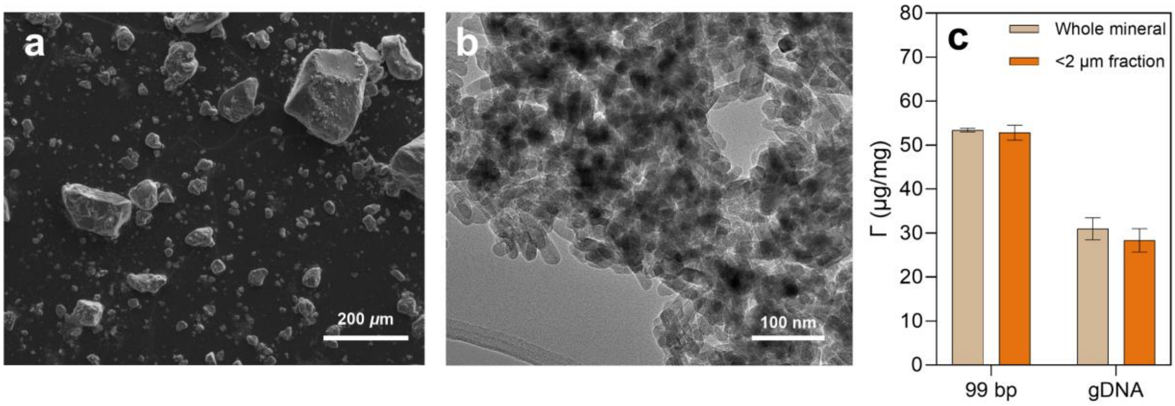

Figure S10: Effect of hydroxyapatite particle size on adsorption. **(a)** Scanning electron micrograph of hydroxyapatite, showing mineral particle aggregates up to ~200 μm. These aggregates form during synthesis as the excess surface energy of small nuclei counterbalances the interparticle repulsive forces. **(b)** Transmission electron micrograph displaying primary particles <100 nm. Before imaging, the mineral was suspended in ethanol and ultrasonicated at 35 kHz for 3 minutes, following the same procedure used prior to adsorption experiments. **(c)** Adsorption of 99 bp DNA and gDNA onto two hydroxyapatite samples with different particle size distributions: one with large aggregates up to 200 μm and another size-fractionated to <2 μm by gravitational settling. Adsorption extent was similar for both DNA polymers across the two hydroxyapatite samples, indicating that the aggregation state did not influence DNA adsorption. Bar heights and error bars represent the mean and standard deviation of triplicates run in parallel.

Section S7: Extended adsorption data

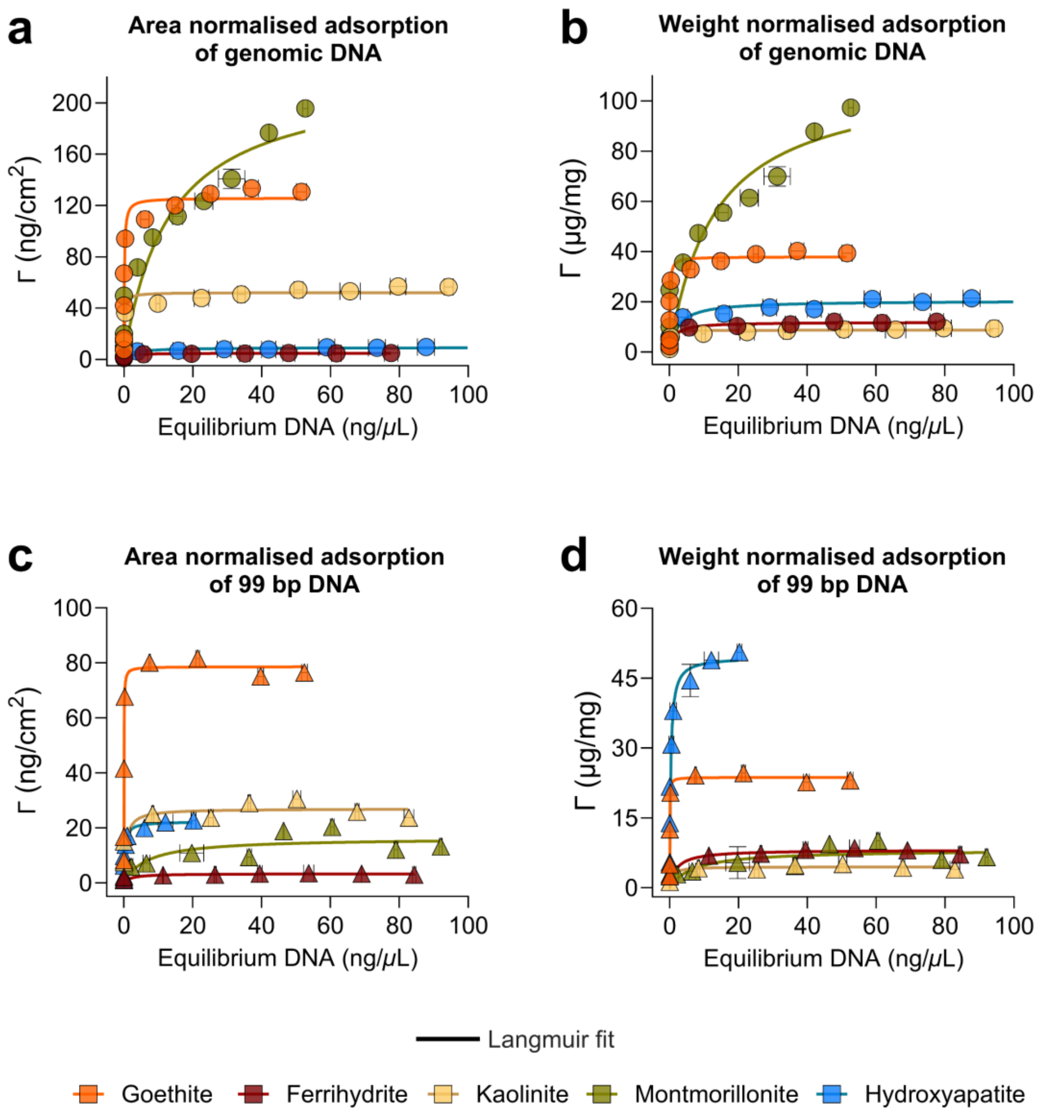

Figure S11: Detailed adsorption isotherms. Adsorption isotherms of 99 bp DNA and gDNA, normalized by mineral surface area and weight, are presented on a single panel for comparison.

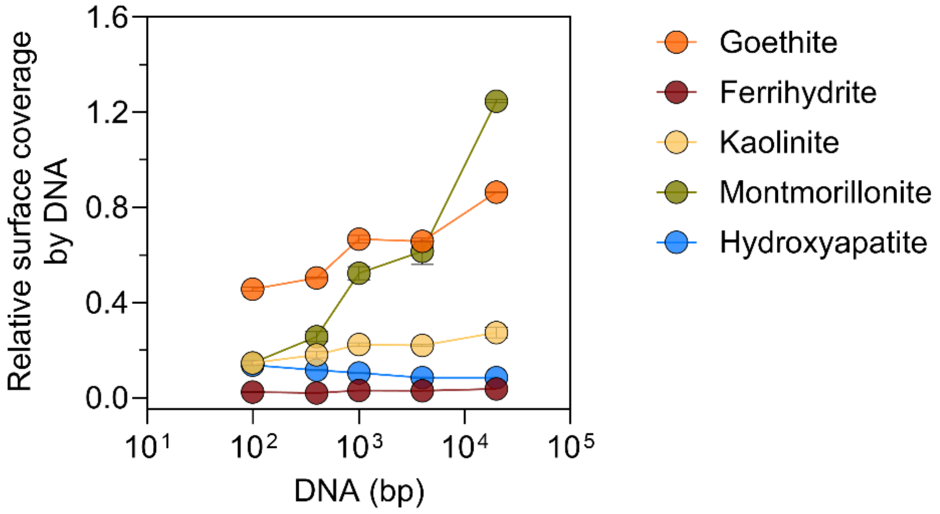

Figure S12: Relative surface coverage of adsorbed DNA on minerals. Surface area occupied by adsorbed DNA polymers at maximum adsorption capacity, relative to the available mineral surface area, was estimated based on data from Figure 2 (main text), assuming DNA to be a cylindrical molecule lying flat on the mineral surface, with a diameter of 2 nm and a base pair length of 0.34 nm. Values below 1 indicate incomplete surface coverage, suggesting that multilayer adsorption is unlikely—except for gDNA on montmorillonite, where values exceed 1.

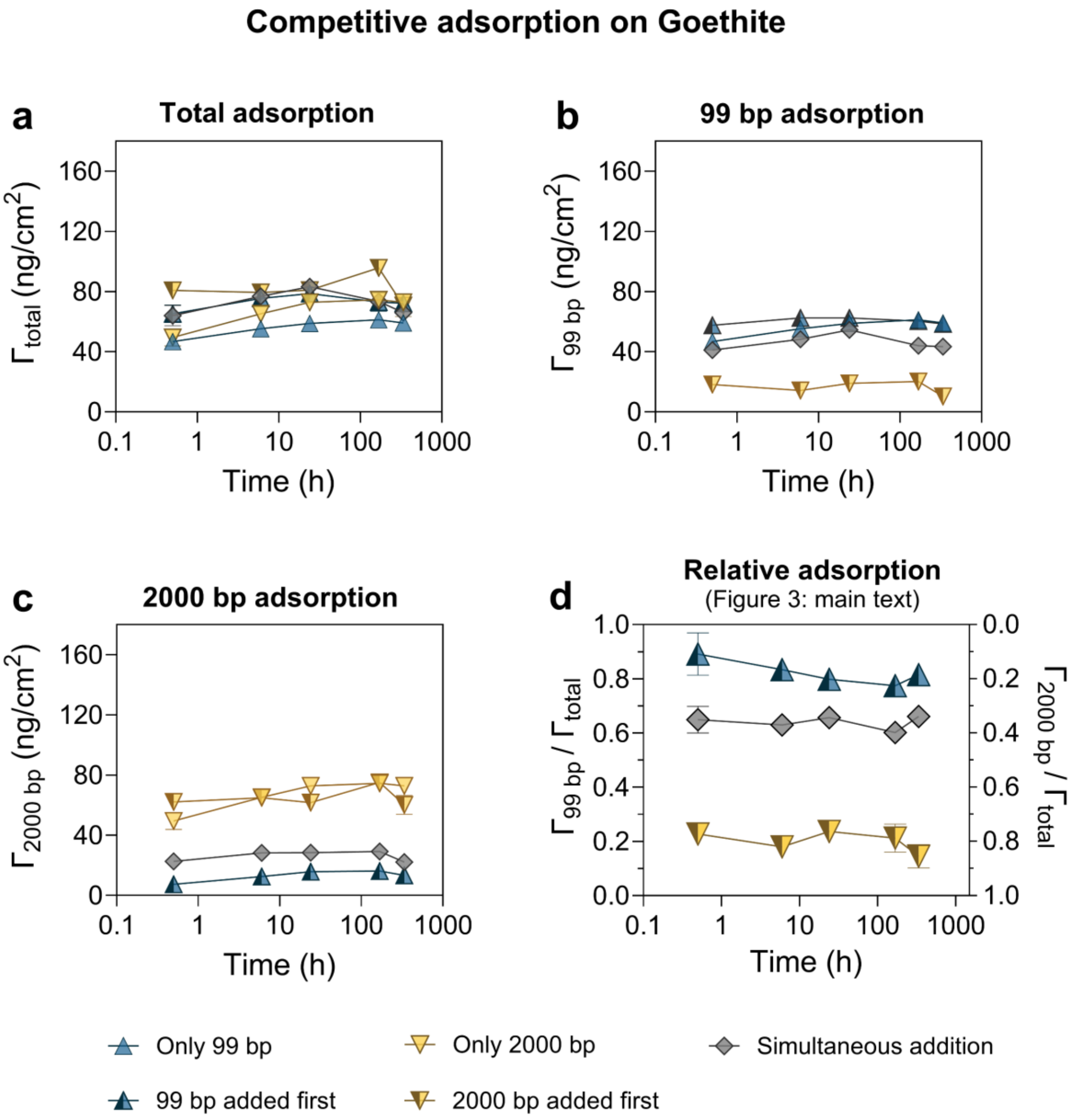

Figure S13.1: Competitive adsorption on Goethite (a) Total adsorbed DNA during competitive adsorption experiments (including simultaneous and sequential addition of 99 and 2000 bp DNA) and experiments with only a single DNA polymer (either 99 bp or 2000 bp) as controls. **(b)** Adsorption of only 99 bp DNA out of the total adsorbed amount. **(c)** Adsorption of only 2000 bp DNA out of the total adsorbed amount. **(d)** relative adsorption of DNA polymers during competitive adsorption experiments. The initial DNA concentration of each DNA polymer in a single DNA system and in competitive systems (after adding the second polymer) was 15 ng/μL, sufficient to reach the respective adsorption plateau on the adsorption isotherm.

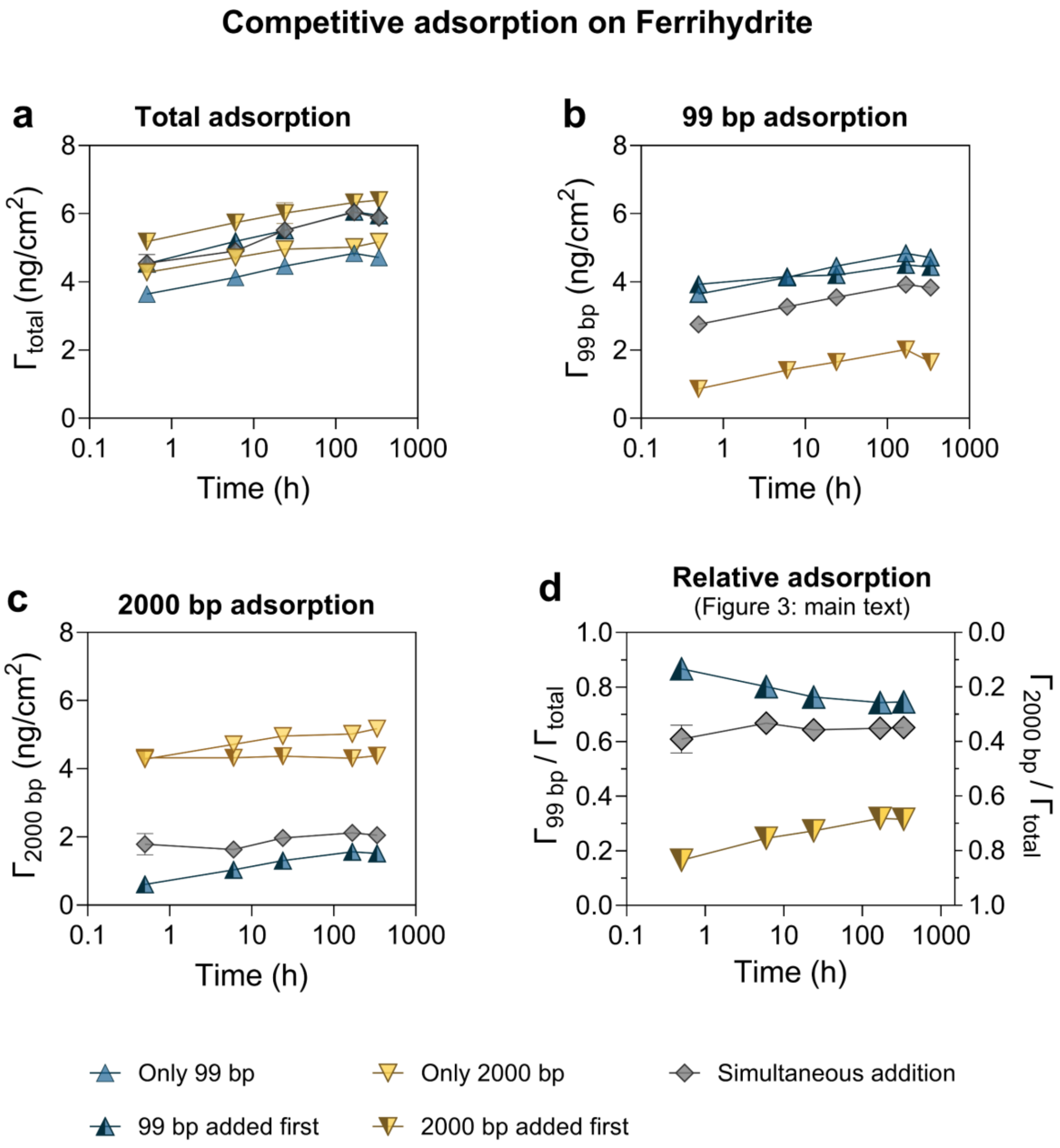

Figure S13.2: Competitive adsorption on Ferrihydrite (a) Total adsorbed DNA during competitive adsorption experiments (including simultaneous and sequential addition of 99 and 2000 bp DNA) and experiments with only a single DNA polymer (either 99 bp or 2000 bp) as controls. **(b)** Adsorption of only 99 bp DNA out of the total adsorbed amount. **(c)** Adsorption of only 2000 bp DNA out of the total adsorbed amount. **(d)** relative adsorption of DNA polymers during competitive adsorption experiments. The initial DNA concentration of each DNA polymer in a single DNA system and in competitive systems (after adding the second polymer) was 15 ng/μL, sufficient to reach the respective adsorption plateau on the adsorption isotherm.

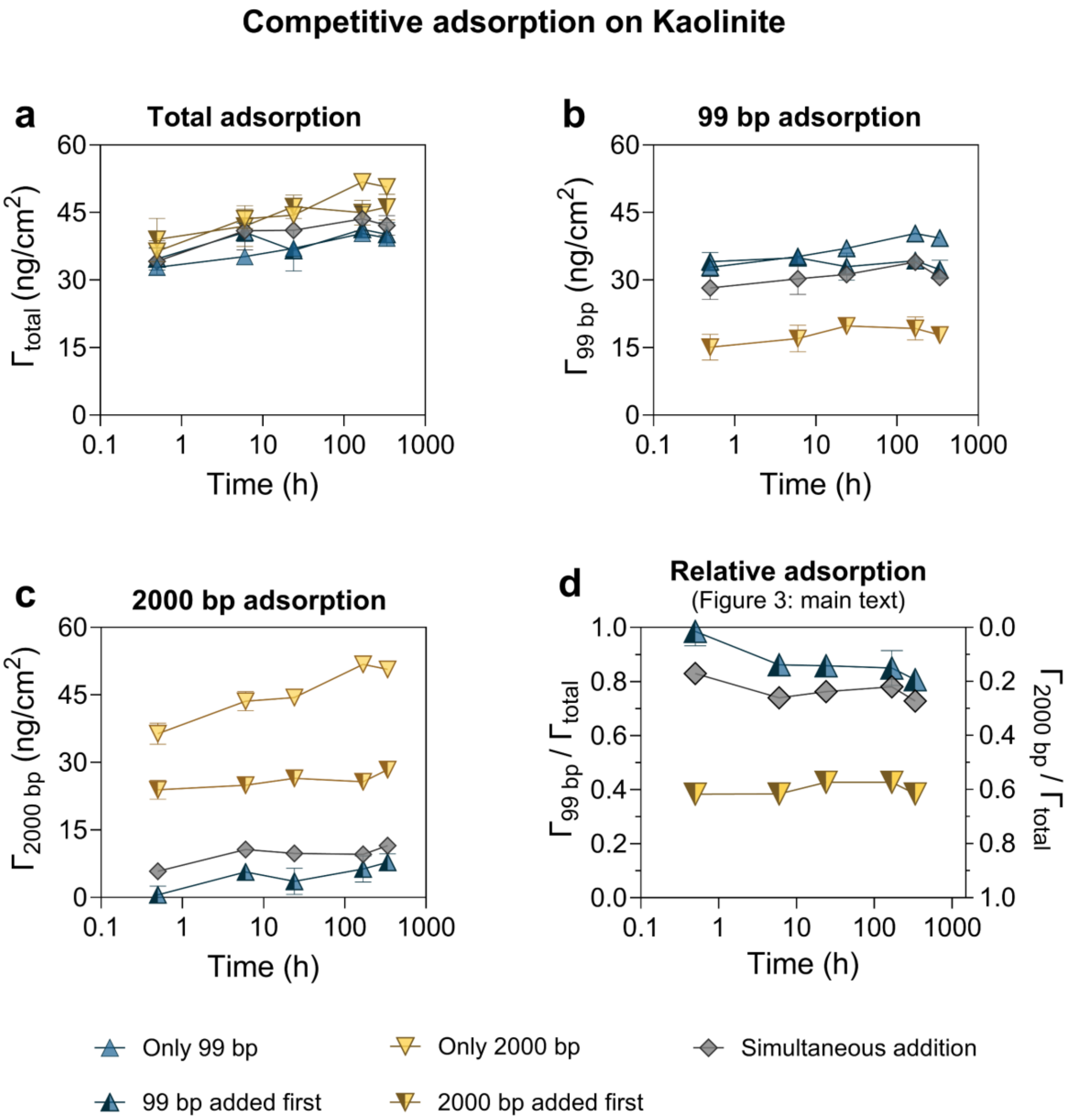

Figure S13.3: Competitive adsorption on Kaolinite (a) Total adsorbed DNA during competitive adsorption experiments (including simultaneous and sequential addition of 99 and 2000 bp DNA) and experiments with only a single DNA polymer (either 99 bp or 2000 bp) as controls. **(b)** Adsorption of only 99 bp DNA out of the total adsorbed amount. **(c)** Adsorption of only 2000 bp DNA out of the total adsorbed amount. **(d)** relative adsorption of DNA polymers during competitive adsorption experiments. The initial DNA concentration of each DNA polymer in a single DNA system and in competitive systems (after adding the second polymer) was 15 ng/μL, sufficient to reach the respective adsorption plateau on the adsorption isotherm.

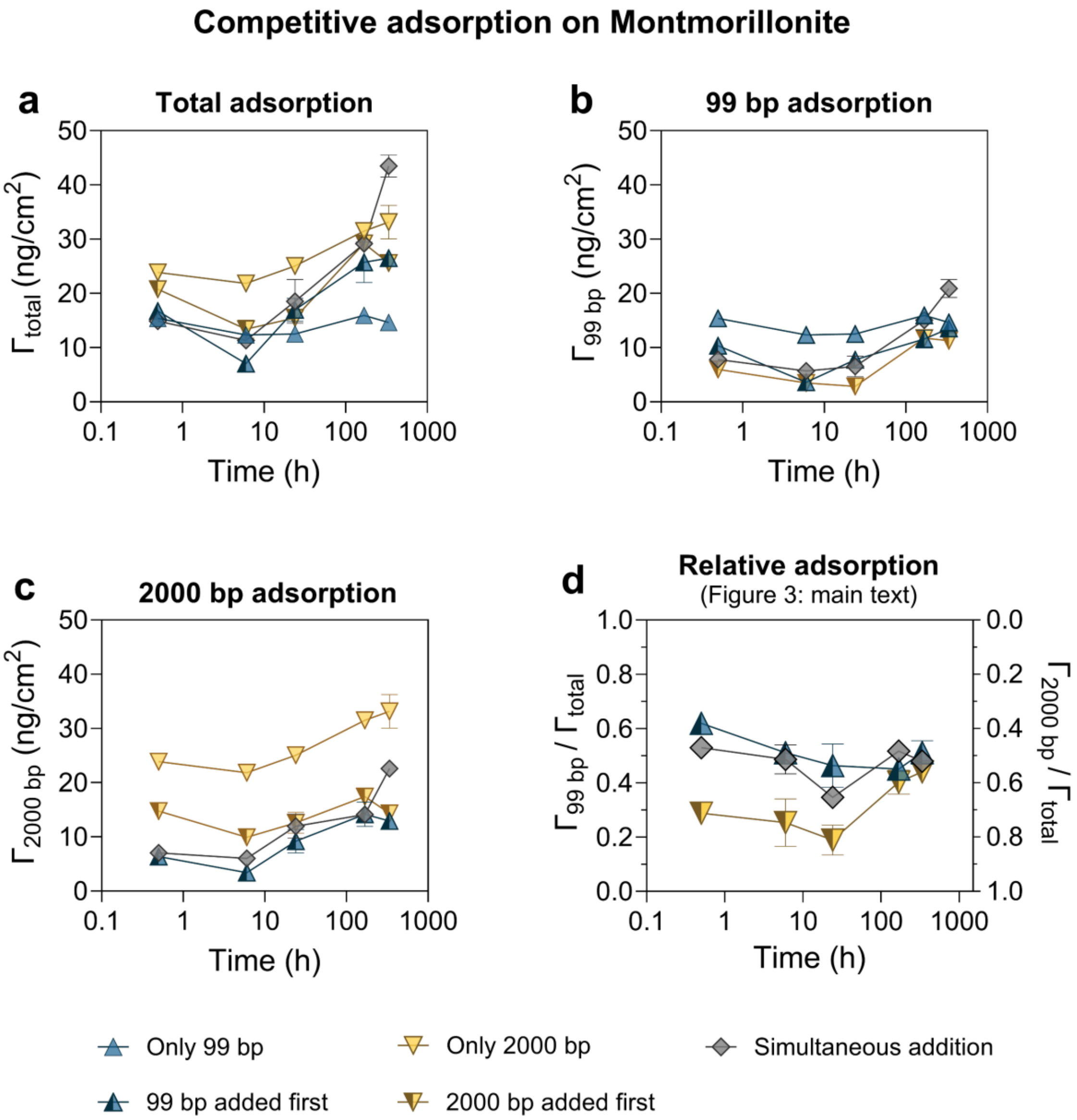

Figure S13.4: Competitive adsorption on Montmorillonite (a) Total adsorbed DNA during competitive adsorption experiments (including simultaneous and sequential addition of 99 and 2000 bp DNA) and experiments with only a single DNA polymer (either 99 bp or 2000 bp) as controls. **(b)** Adsorption of only 99 bp DNA out of the total adsorbed amount. **(c)** Adsorption of only 2000 bp DNA out of the total adsorbed amount. **(d)** relative adsorption of DNA polymers during competitive adsorption experiments. The initial DNA concentration of each DNA polymer in a single DNA system and in competitive systems (after adding the second polymer) was 15 ng/μL, sufficient to reach the respective adsorption plateau on the adsorption isotherm.

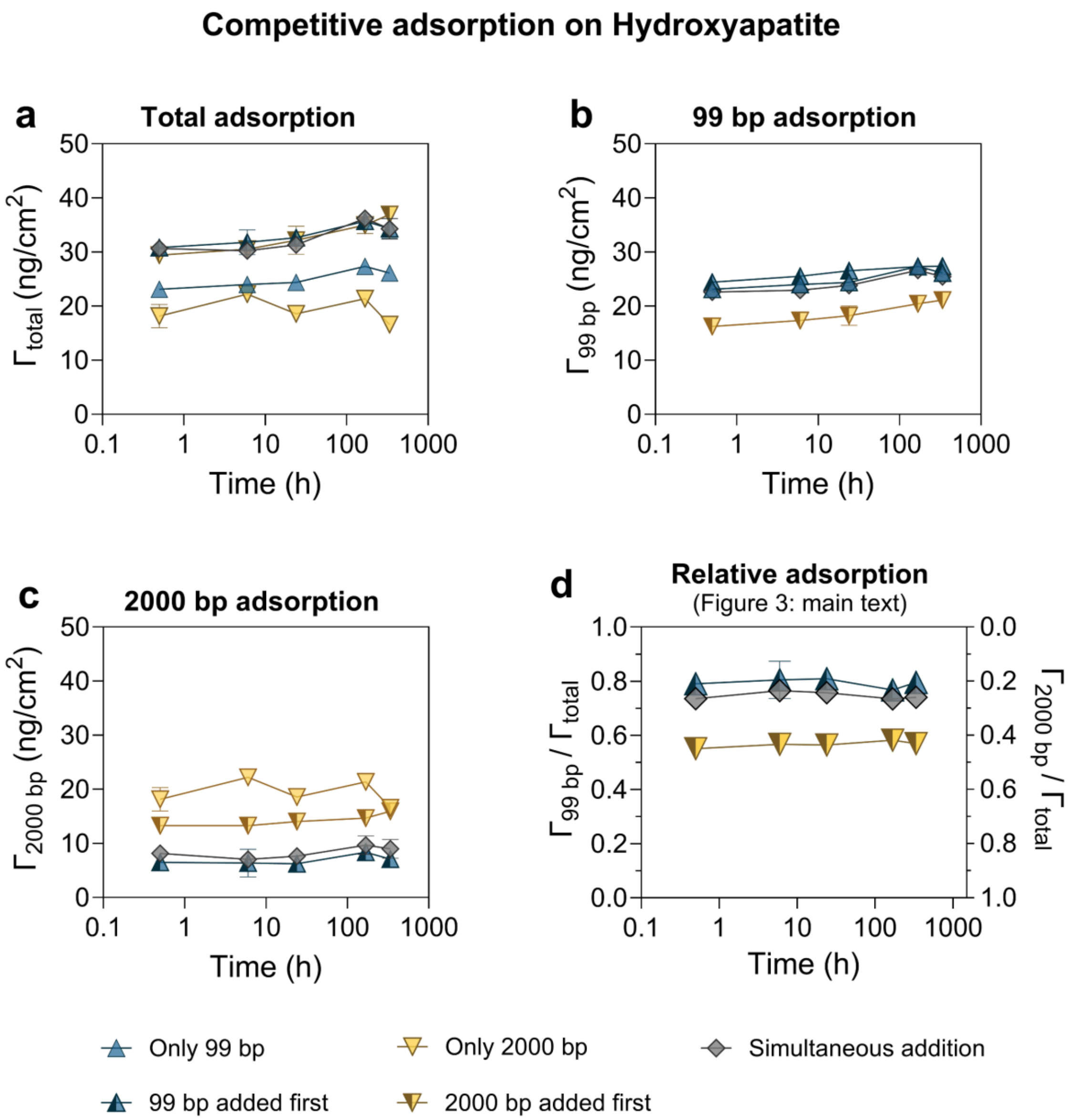

Figure S13.5: Competitive adsorption on Hydroxyapatite (a) Total adsorbed DNA during competitive adsorption experiments (including simultaneous and sequential addition of 99 and 2000 bp DNA) and experiments with only a single DNA polymer (either 99 bp or 2000 bp) as controls. **(b)** Adsorption of only 99 bp DNA out of the total adsorbed amount. **(c)** Adsorption of only 2000 bp DNA out of the total adsorbed amount. **(d)** relative adsorption of DNA polymers during competitive adsorption experiments. The initial DNA concentration of each DNA polymer in a single DNA system and in competitive systems (after adding the second polymer) was 15 ng/μL, sufficient to reach the respective adsorption plateau on the adsorption isotherm.

References

(1) John Tymoczko; Jeremy M. Berg; Gregory J. Gatto Jr.; Lubert Stryer. *Biochemistry: A Short Course*, 4th ed.; W.H. Freeman and Company Ltd., 2018.

(2) Lukacs, G. L.; Haggie, P.; Seksek, O.; Lechardeur, D.; Freedman, N.; Verkman, A. S. Size-Dependent DNA Mobility in Cytoplasm and Nucleus. *J. Biol. Biochem*. **2000**, *275* (3), 1625–1629.

(3) Schwertmann, U.; Cornell, R. M. *Iron Oxides in the Laboratory: Preparation and Characterization*, 2nd ed.; Wiley-VCH, 2000.

(4) Van Groeningen, N.; Christl, I.; Kretzschmar, R. The Effect of Aeration on Mn(II) Sorbed to Clay Minerals and Its Impact on Cd Retention. *Environ. Sci. Technol.* **2021**, *55* (3), 1650–1658.

(5) Li, Y.; Wang, Y.; Li, Y.; Luo, W.; Jiang, J.; Zhao, J.; Liu, C. Controllable Synthesis of Biomimetic Hydroxyapatite Nanorods with High Osteogenic Bioactivity. *ACS Biomater. Sci. Eng.* **2019**, *6* (1), 320–328.

(6) Doebelin, N.; Kleeberg, R. Profex: A Graphical User Interface for the Rietveld Refinement Program BGMN. *J. Appl. Crystallogr.* **2015**, *48* (5), 1573–1580.

(7) Chipera, S. J.; Bish, D. L. Baseline Studies of the Clay Minerals Society Source Clays: Powder X-Ray Diffraction Analyses. *Clays Clay Miner.* **2001**, *49* (5), 398–409.

(8) Millman, E.; Chatterjee, A.; Parker, K. M.; Catalano, J. G. Cation Exchange to Montmorillonite Induces Selective Adsorption of Amino Acids. *Geochim. Cosmochim. Acta* **2024**, *372*, 181–195.

(9) *Physical and Chemical Data of Source Clays – The Clay Minerals Society*. (https://www.clays.org/sourceclays_data/)

(10) Kumar, N.; Lezama Pacheco, J.; Noël, V.; Dublet, G.; Brown, G. E. Sulfidation Mechanisms of Fe(III)-(Oxyhydr)Oxide Nanoparticles: A Spectroscopic Study. *Environ. Sci. Nano.* **2018**, *5* (4), 1012–1026.

(11) Hao, W.; Flynn, S. L.; Alessi, D. S.; Konhauser, K. O. Change of the Point of Zero Net Proton Charge (PHPZNPC) of Clay Minerals with Ionic Strength. *Chem. Geol.* **2018**, *493*, 458–467.

(12) Borden, D.; Giese, R. F. Baseline Studies of the Clay Minerals Society Source Clays: Cation Exchange Capacity Measurements by the Ammonia-Electrode Method. *Clays. Clay. Miner.* **2001**, *49* (5), 444–445.

(13) Bell, L. C.; Posner, A. M.; Quirk, J. P. Surface Charge Characteristics of Hydroxyapatite and Fluorapatite. *Nature* **1972**, *239* (5374), 515–517.
